# High efficiency wideband printed monopole antenna with enhanced gain using artificial magnetic conductor surface

**DOI:** 10.1038/s41598-025-99233-z

**Published:** 2025-05-12

**Authors:** A. E. Farahat, May AboEl-Hassan, K. F. A. Hussein

**Affiliations:** Electronics Research Institute (ERI), Cairo, Egypt

**Keywords:** Artificial magnetic conducting surface, Frequency selective surface, High-gain antenna, Periodic structure., Electrical and electronic engineering, Scientific data

## Abstract

Combining the benefits of a low profile, high gain, high efficiency, and wideband operation in a planar antenna presents a significant challenge for antenna designers. Low-profile wideband antennas often suffer from low gain. This study introduces a compact wideband artificial magnetic conducting surface (AMCS) positioned behind a wideband omnidirectional antenna to enhance its gain across the operational frequency range. This integration allows the radiating structure to achieve both high gain and wideband functionality in a single design. In this research, a wideband planar monopole printed antenna is developed to function as an omnidirectional radiator, delivering excellent impedance matching and radiation efficiency across the frequency range of 3.9–7.2 GHz (60% bandwidth) in free space. The free-standing antenna dimensions are 30 mm × 20 mm (0.39 λ_o_ × 0.3 λ_o_), where λ_o_ corresponds to the lowest operating frequency of the antenna). It exhibits a gain ranging from 2 dBi to 4.5 dBi over this frequency band. To improve gain, a wideband AMCS is designed, consisting of just 3 × 3 unit cells with overall dimensions of 9 × 9 cm (1.1 λ_o_ × 1.1 λ_o_). The AMCS is placed parallel to the planar antenna at a distance of 1.75 cm behind it. The gain of the AMCS-backed antenna reaches up to 9 dBi without compromising bandwidth or impedance matching. Furthermore, the radiation efficiency remains above 98% across the operational band of 3.6–7.2 GHz (66% bandwidth). The wideband antenna and AMCS are fabricated to experimentally validate the performance of the AMCS-based antenna. Measurements of impedance matching, gain, and radiation efficiency demonstrate close alignment with simulation results, confirming the effectiveness of the proposed design.

## Introduction

In the current and forthcoming generations of wireless communication, the demand for low-profile, wideband, and high-gain antennas has become a key focus for advancing high-performance communication systems. Consequently, low-profile, high-efficiency wideband antennas are essential for practical applications and continue to be a significant area of research.

In general, metasurfaces can create materials with unusual electromagnetic properties, such as negative permittivity and permeability^1,2^. An artificial magnetic conducting surfaces (AMCS) belongs to the category of metasurfaces. One effective approach to enhance bandwidth and gain is through the use of, which modify the antenna’s electromagnetic properties. Performance can also be optimized by incorporating frequency-selective surfaces (FSS) or multilayer configurations. By carefully designing the unit cell of an FSS, it is possible to construct a structure that blocks unwanted frequencies while enabling the transmission of desired ultra-wideband (UWB) signals. The AMCS-based antennas are required for many applications including 5G and future wireless networks, fixed satellite services, mobile satellite communication, Airborne and Ground-based Radar, and automotive radar (for autonomous vehicles).

FSSs are resonant structures with frequency responses influenced by their material properties, geometry, and the spacing between unit cells. These properties allow for precise control of transmitted electromagnetic waves, thereby enhancing the antenna’s operating bandwidth and gain^3,4^. Additionally, FSSs can mitigate issues caused by nearby metallic surfaces, such as reflections and distortions in the antenna’s radiation pattern. Advances in AMCS technology are necessary to develop low-profile antennas with unidirectional radiation and broad impedance bandwidth. Researchers have demonstrated the potential to significantly expand the reflection phase bandwidth^5^.

Various monopole and dipole antennas based on AMCS structures have been reported in^6–8^. However, these designs often suffer from limited reflection phase bandwidth, restricting their impedance bandwidth to 5–10%. To improve wideband antenna performance, a wideband AMC structure with an expanded reflection phase bandwidth (within − 90° to + 90°) is required.

Recent studies have introduced innovative AMCS geometries as reflectors to enhance the radiation performance of wideband antennas with reduced profiles^9–18^. For example, in^16^, an AMCS reflector was placed beneath an ultra-wideband MIMO antenna, achieving a 2.3 dBi gain enhancement. Similarly, an AMCS-backed antenna in^17^ provided a gain exceeding 8 dBi but was constrained by its significant spatial requirements. Therefore, developing a low-profile, cost-effective, lightweight wideband antenna with improved gain and high efficiency is achievable through a well-designed AMCS.

This study presents a novel wideband planar antenna design incorporating a wideband AMCS to boost antenna gain across all operating frequencies while maintaining the bandwidth. Section Design of the wideband free-standing antenna outlines the design of the free-standing wideband antenna. Section Parametric study for optimum design of the free-standing antenna discusses parameter sweep simulations to optimize the antenna’s dimensions. Section Antenna fabrication and measurement describes the fabrication process and the practical measurements used to evaluate its performance. Section Radiation characteristics of the free-standing antenna provides numerical results detailing the radiation characteristics of the free-standing antenna. Section Design of the AMCS for wideband operation focuses on the design and numerical analysis of the proposed AMCS. Section Fabrication and measurement of the antenna over the AMC surface presents experimental evaluations of the AMCS-backed antenna’s overall performance. Section Comparison with other AMCS-backed antenna designs compares the proposed design with other published work, offering a comparative evaluation. Finally, Sect. Conclusion concludes the study with key findings.

## Design of the wideband free-standing antenna

**Fig. 1:**
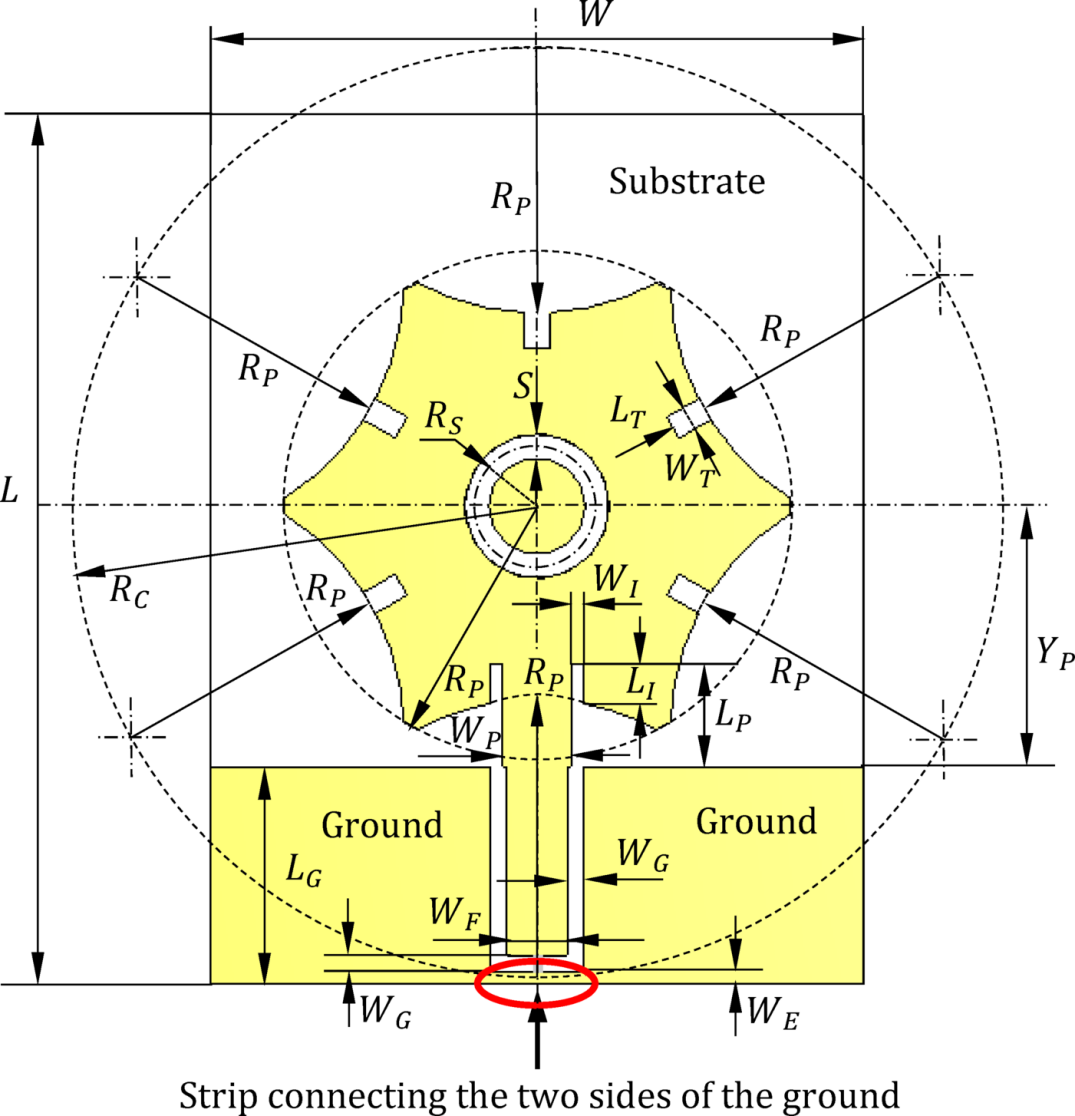
Geometry and design parameters of the star-shaped monopole patch antenna.

The design of the free-standing antenna is depicted in Fig. 1. It features a star-shaped planar monopole patch antenna fed by a coplanar waveguide (CPW). The radiating patch and the CPW feeder are printed on one side of the substrate, leaving the opposite side blank. The substrate material is Rogers RO4003 C with dielectric constant $\:{\epsilon\:}_{r}=3.55$, loss tangent, $\:\text{tan}\delta\:=\:0.0027$, and thickness $\:h=1.52\:\text{m}\text{m}.$ The star-shaped geometry consists of a central circle and six uniformly distributed surrounding circles of equal radius ($\:{R}_{P}$). The centers of these circles lie on a circle of radius $\:{R}_{C}$ where $\:{R}_{C}<2{R}_{P}$. A circular slot with a radius $\:{R}_{S}$ is positioned at the center of the radiating patch. This slot adjusts the surface current distribution near the center, aiding in input impedance matching and improving radiation characteristics. Optimal performance is achieved by carefully tuning $\:{R}_{S}$. Additionally, five uniformly distributed slits are incorporated along the patch perimeter, with each slit having a length $\:{L}_{T}$ and width $\:{W}_{T}$. These slits help modify the current distribution along the patch edges, enhancing impedance matching and antenna efficiency. To enhance the impedance matching bandwidth, a strip extension of width $\:{W}_{P}$ connects the radiating patch to the CPW’s central conductor. The width of the central strip ($\:{W}_{F}$) and the side slots ($\:{W}_{G}$) are optimized to achieve the desired frequency bandwidth and operational range. Furthermore, two slits are added along the sides of the feed line extension to improve impedance matching. These slits have a length $\:{L}_{I}$ and width $\:{W}_{I}$.

Extensive parameter sweeps were conducted using the CST simulator to optimize the antenna’s operational bandwidth and radiation efficiency. The final design parameters, shown in Fig. 1, are summarized in Table 1. Section Parametric study for optimum design of the free-standing antenna provides simplified examples and further discussion of these parameter sweeps.

**Table 1:** Dimensions of the star-shaped monopole patch antenn a whose geometry is shown in Fig. 1.

Parameter	$\:L$	$\:{L}_{G}$	$\:{L}_{I}$	$\:{L}_{P}$	$\:{L}_{T}$	$\:{R}_{C}$	$\:{R}_{P}$	$\:{R}_{S}$	$\:S$
Value (mm)	$\:30$	$\:9$	$\:1.67$	$\:4.3$	$\:1.51$	$\:19.5$	$\:10.5$	$\:2.5$	$\:1.0$
Parameter	$\:{Y}_{P}$	$\:W$	$\:{W}_{G}$	$\:{W}_{E}$	$\:{W}_{F}$	$\:{W}_{I}$	$\:{W}_{P}$	$\:{W}_{T}$	$\:h$
Value (mm)	$\:10.8$	$\:23$	$\:0.7$	$\:0.5$	$\:2.5$	$\:0.5$	$\:2.8$	$\:1.0$	$\:1.52$

## Parametric study for optimum design of the free-standing antenna

This section provides six examples of parameter sweeps performed via simulation to conduct a comprehensive parametric study and determine optimal geometrical parameters. The analysis focuses on varying parameters $\:{R}_{P}$, $\:{R}_{C}$, $\:{Y}_{P}$, $\:{R}_{S}$, $\:{W}_{F}$, and $\:{W}_{G}$, while keeping the other design parameters fixed as specified in Table 1.

**Fig. 2:**
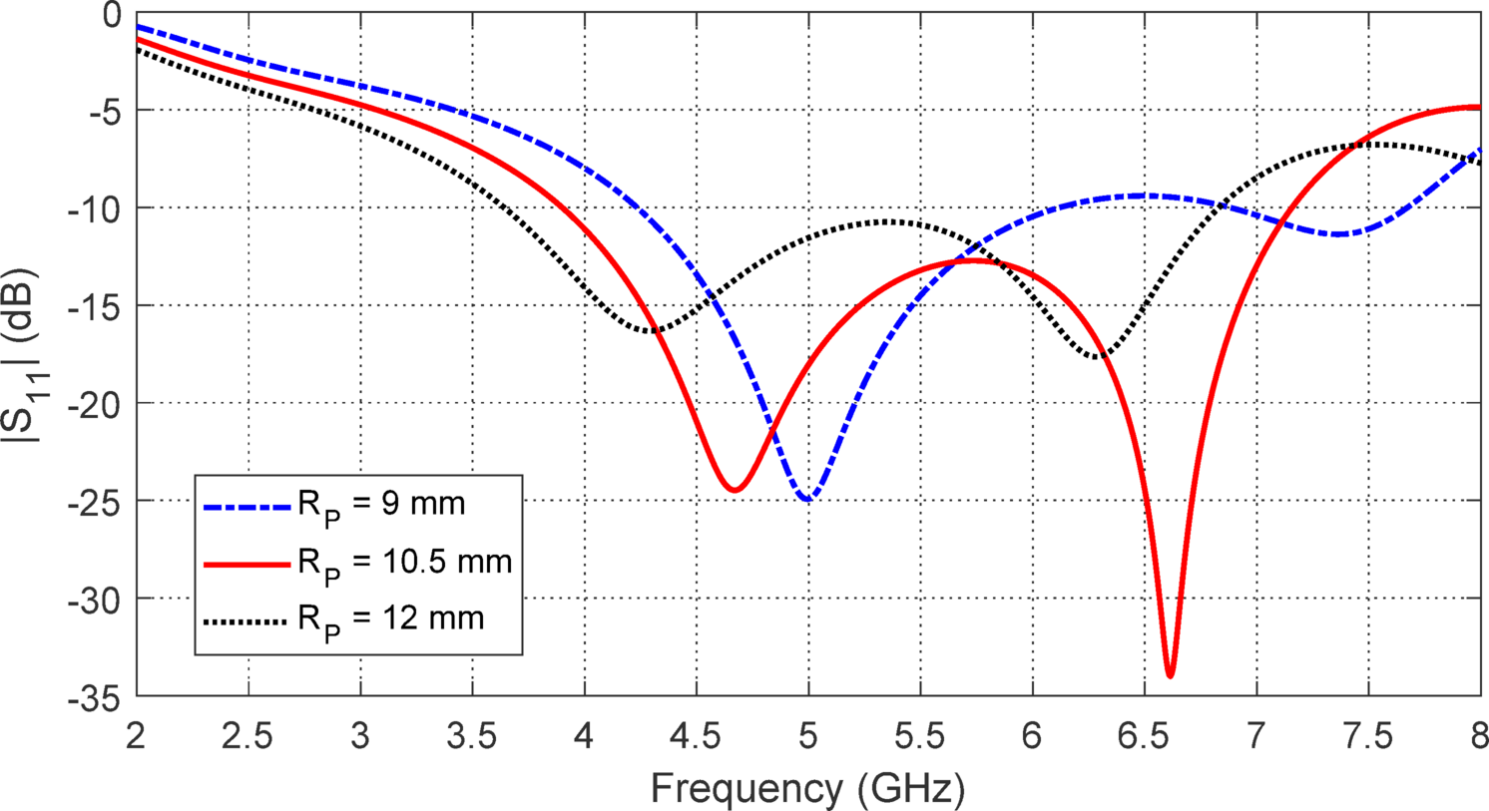
Variation of $\:\left|{S}_{11}\right|$ of the star-shaped antenna with the frequency for different values of $\:{R}_{P}$.

The size of the radiating patch is primarily determined by the radius $\:{R}_{P}$​. Figure 2 illustrates the frequency response of $\:\left|{S}_{11}\right|$ for different $\:{R}_{P}$​ values, showing a significant impact on both the start and end of the operating band (using $\:\left|{S}_{11}\right|<-10\:\text{d}\text{B}$ as the bandwidth criterion). To achieve optimal impedance matching and maximum bandwidth,$\:\:{R}_{P}=10.5\:\text{m}\text{m}$ is selected.

**Fig. 3:**
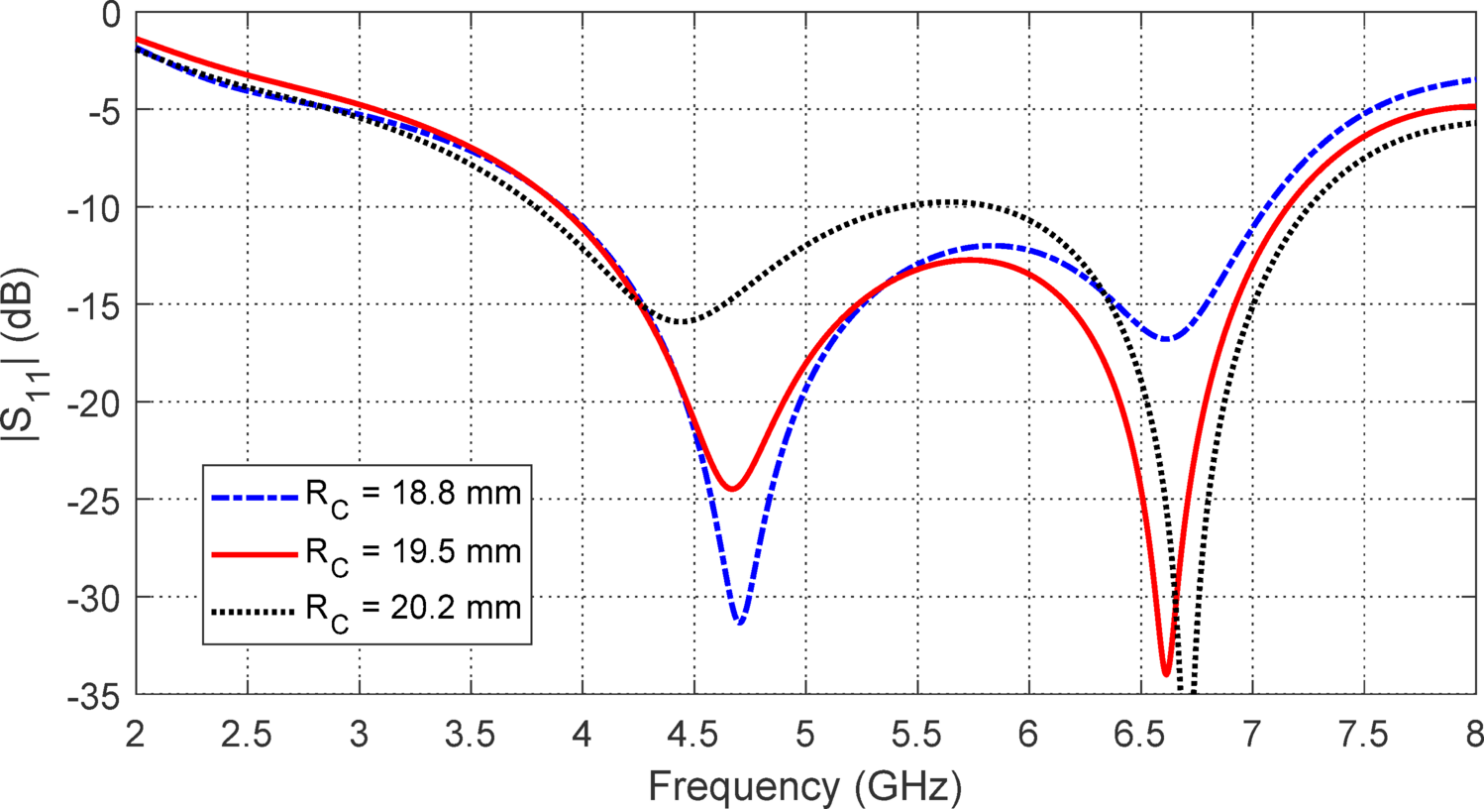
Variation of $\:\left|{S}_{11}\right|$ of the star-shaped antenna with the frequency for different values of $\:{R}_{C}$.

The star-shaped patch is created by removing six circular segments from a central circular patch using six surrounding cutting circles. The centers of these cutting circles are positioned along the circumference of a circle with radius $\:{R}_{C}$​, as illustrated in Fig. 1. The radius $\:{R}_{C}$​ influences the patch area and, consequently, the operating band. Figure 3 shows that changes in $\:{R}_{C}$​ have a minor impact on the start and end frequencies of the operating band ($\:\left|{S}_{11}\right|<-10\:\text{d}\text{B}$) but significantly affect $\:\left|{S}_{11}\right|$ value. The optimal dimension, $\:{R}_{C}=19.5\:\text{m}\text{m}$, provides the widest operating frequency band.

**Fig. 4:**
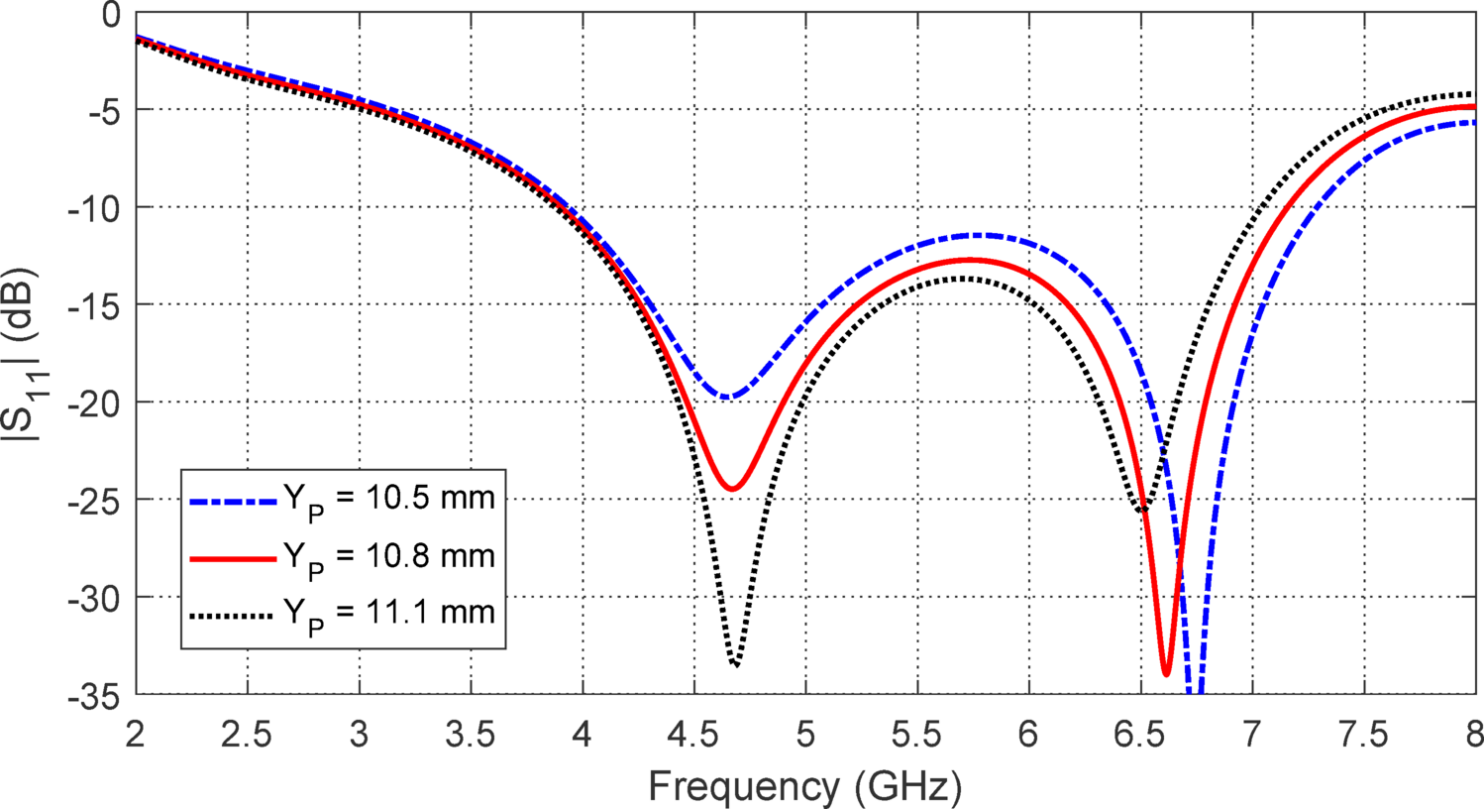
Variation of $\:\left|{S}_{11}\right|$ of the star-shaped antenna with the frequency for different values of $\:{Y}_{P}$.

Adjusting the distance $\:{Y}_{P}$ between the end of the CPW feeder and the center of the radiating patch influences $\:\left|{S}_{11}\right|$ frequency response within the $2-8$ GHz range, as depicted in Fig. 4. While the start frequency remains largely unaffected by changes in $\:{Y}_{P}$, the end frequency shows significant sensitivity. To ensure optimal impedance matching across the broadest frequency band, $\:{Y}_{P}=12.3\:\text{m}\text{m}$.

**Fig. 5:**
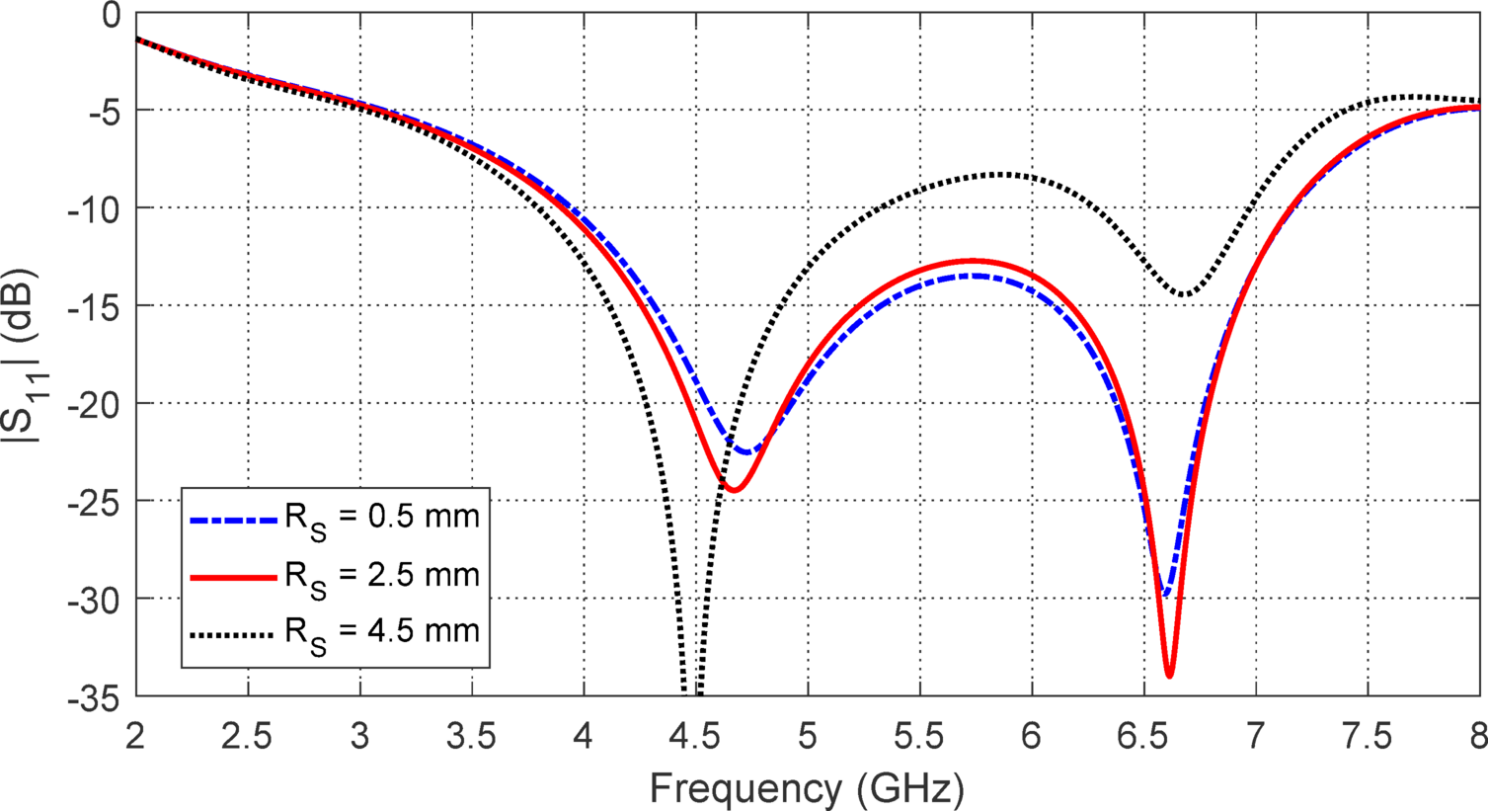
Variation of $\:\left|{S}_{11}\right|$ of the star-shaped antenna with the frequency for different values of $\:{R}_{S}$.

The effect of varying the radius $\:{R}_{S}$​ of the circular centerline of the ring slot at the patch’s center on the frequency response of $\:\left|{S}_{11}\right|$ is illustrated in Fig. 5. The results indicate that $\:{R}_{S}=2.5\:\text{m}\text{m}$ achieves the widest frequency band with optimal impedance matching.

**Fig. 6:**
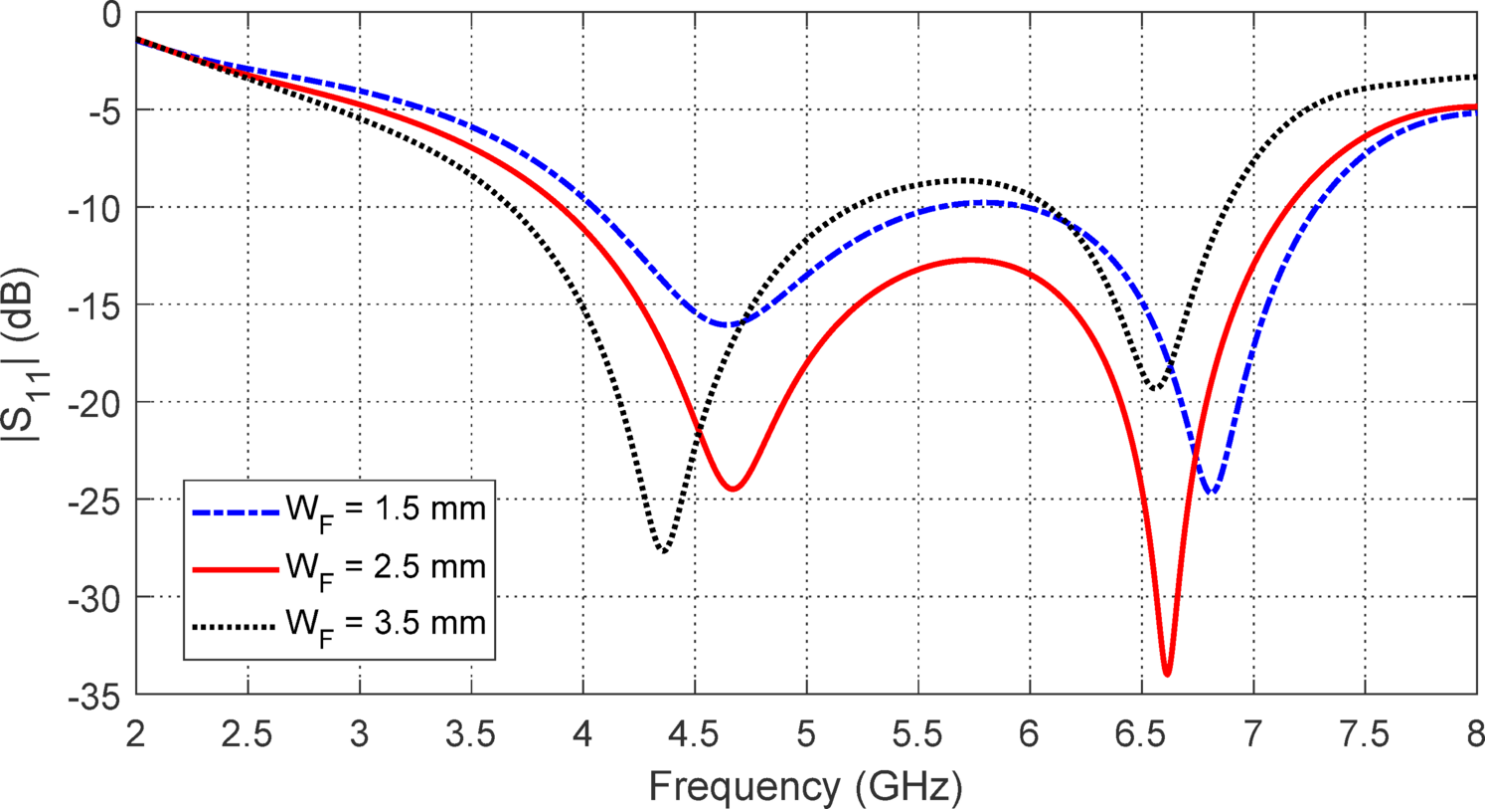
Variation of $\:\left|{S}_{11}\right|$ with the frequency of the proposed antenna, shown in Fig. 1, for different values of $\:{W}_{F}$. The other parameters are as given in Table 1.

Figure 6 shows how varying the width $\:{W}_{F}$ of the central conductor in the CPW feeder impacts the $\:\left|{S}_{11}\right|$ frequency response. This parameter significantly influences both the start and end frequencies of the impedance matching band. Optimal impedance matching and the widest bandwidth are achieved with $\:{W}_{F}=2.5\:\text{m}\text{m}$.

**Fig. 7:**
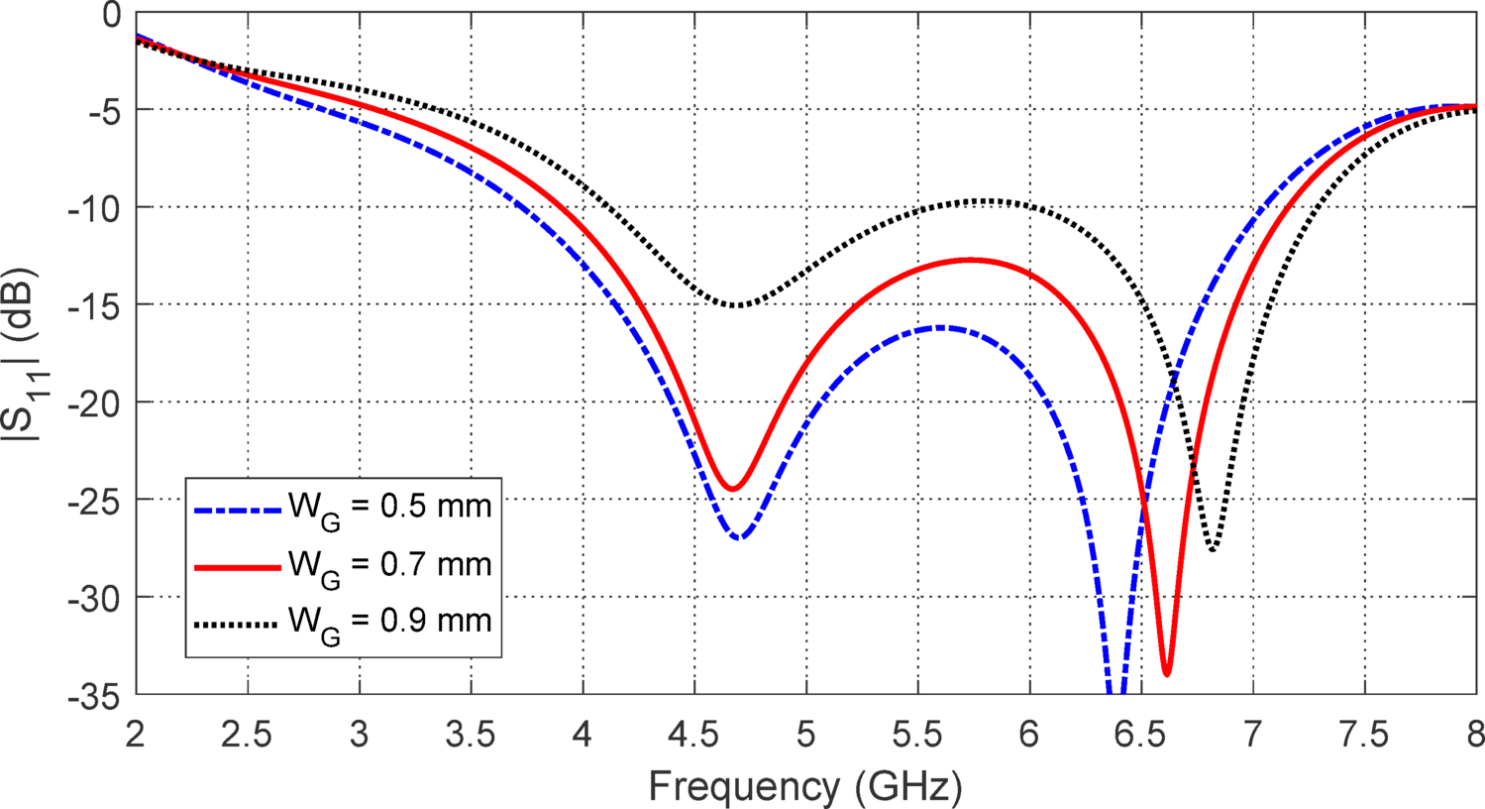
Variation of $\:\left|{S}_{11}\right|$ with the frequency of the proposed antenna, shown in Fig. 1, for different values of $\:{W}_{G}$. The other parameters are as given in Table 1.

Figure 7 shows how varying the width $\:{W}_{G}$ of the side slots in the CPW feeder impacts the $\:\left|{S}_{11}\right|$ frequency response. This parameter significantly influences both the start and end frequencies of the impedance matching band. Optimal impedance matching and the widest bandwidth are achieved with $\:{W}_{G}=0.7\:\text{m}\text{m}$.

Figure 7 illustrates the effect of changing the width $\:{W}_{G}$​ of the side slots in the CPW feeder on the $\:\left|{S}_{11}\right|$ frequency response. As shown in the figure, this parameter plays a crucial role in determining both the start and end frequencies of the impedance matching band. The widest bandwidth and optimal impedance matching are achieved when $\:{W}_{G}=0.7\:\text{m}\text{m}$.

A comprehensive parametric study of the remaining geometrical parameters shown in Fig. 1 was conducted to determine the final antenna dimensions listed in Table 1.

## Antenna fabrication and measurement

A prototype of the free-standing antenna is fabricated for experimental evaluation and subjected to microwave measurements, which are discussed in detail in the following subsection.

### Antenna fabrication

**Fig. 8:**
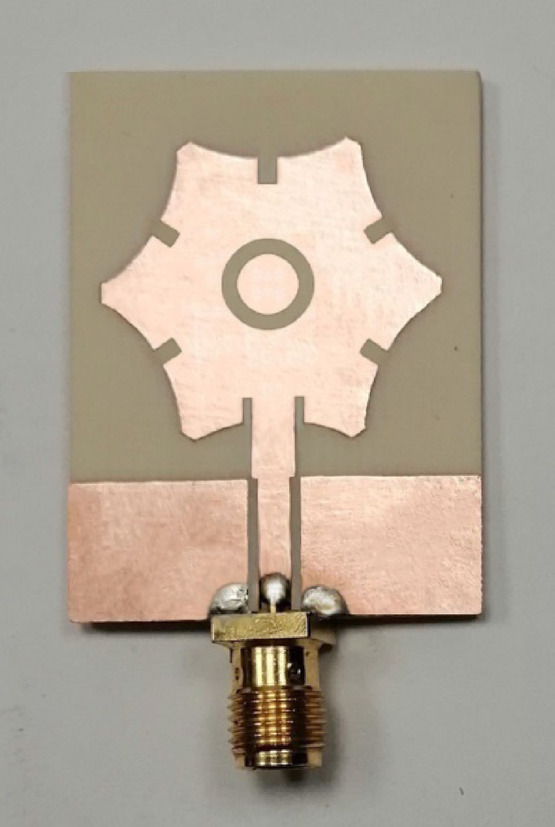
Prototype of the proposed antenna.

The monopole printed antenna depicted in Fig. 1 is fabricated using a single-sided substrate of Roger’s RO4003 C material, characterized by $\:{\epsilon\:}_{r}=3.55$, $\:\text{tan}\delta\:=0.0027$, and thickness $\:h=\:1.52\:\text{m}\text{m}.$ The fabrication process employs conventional lithography techniques. To facilitate measurements, an SMA coaxial connector is attached to the free end of the CPW region. This is achieved by soldering the inner pin of the SMA connector to the central conductor of the CPW and the outer conductor to the side ground planes of the CPW. It is important to note that the bridge strip connecting the two ground planes of the CPW (shown at the bottom of Fig. 1) is removed prior to attaching the SMA launcher. The fabricated antenna, along with the mounted SMA connector, is shown in Fig. 8.

### Antenna bandwidth measurement

**Fig. 9:**
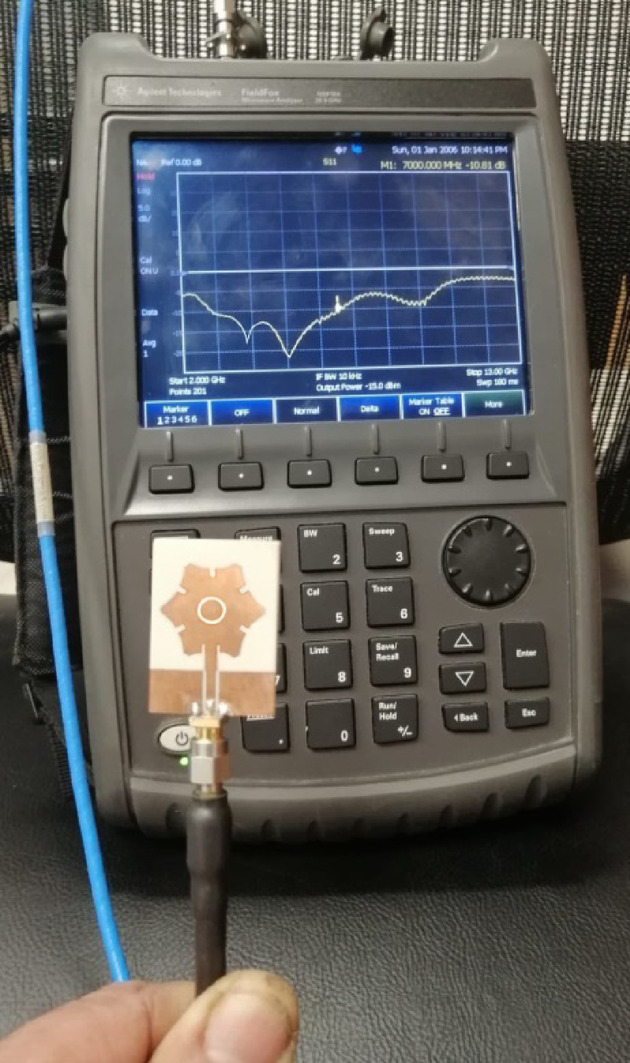
Measurement of $\:{S}_{11}$ over the frequency range $\:2\:$-$\:13\:\text{G}\text{H}\text{z}$.

**Fig. 10:**
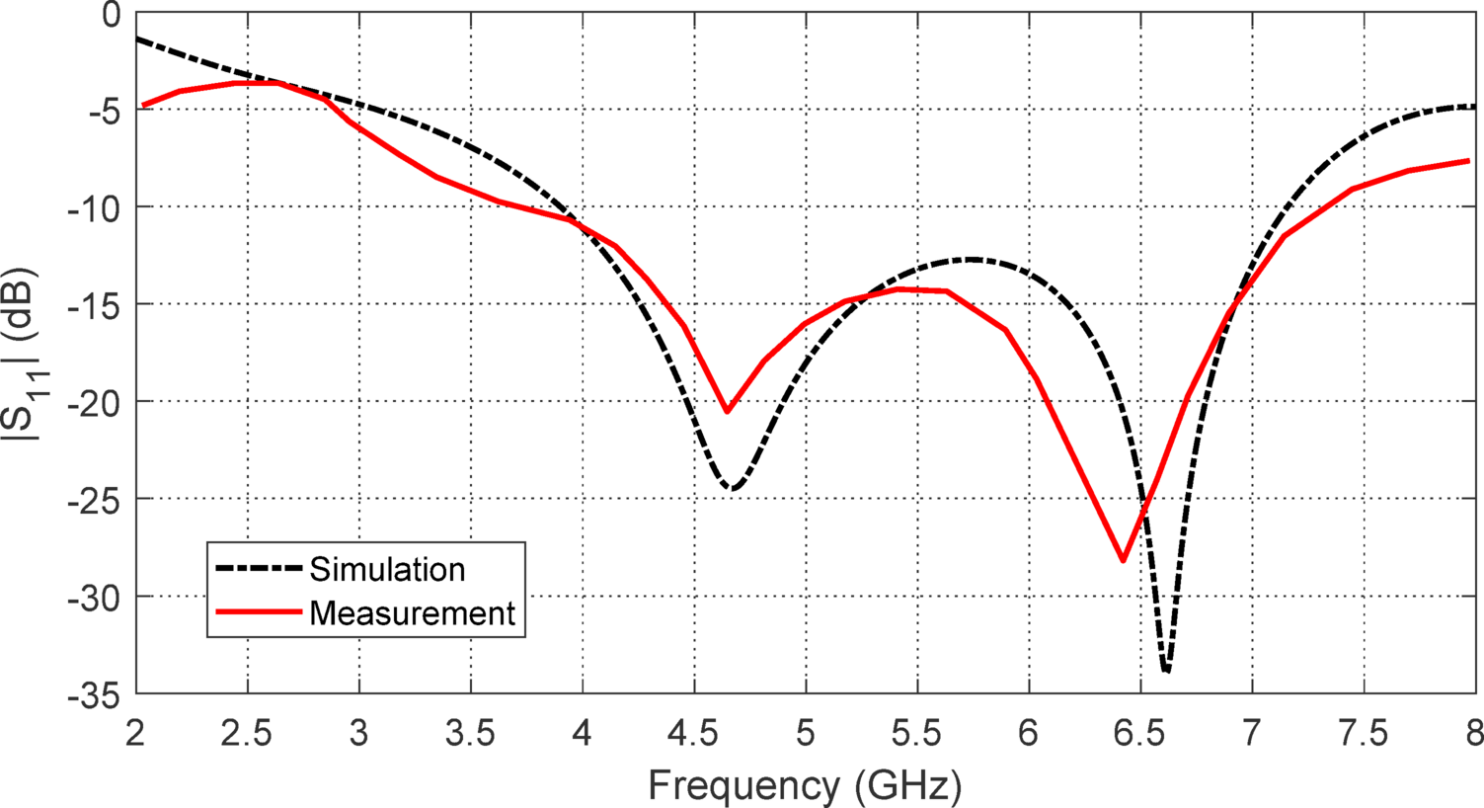
Simulation results and measurements of $\:\left|{S}_{11}\right|$ of the free-standing antenna.

An Agilent N9918 A vector network analyzer (VNA) is utilized to measure $\:{S}_{11}$ at the antenna’s input port. The setup for connecting the experimental antenna to the VNA is shown in Fig. 9, with the measured $\:\left|{S}_{11}\right|$ displayed on the VNA screen across the frequency range of $\:\left|{S}_{11}\right|$ GHz. Figure 10 compares the measured and simulated $\:\left|{S}_{11}\right|$ frequency responses, demonstrating a strong agreement. The impedance matching bandwidth is observed to range from $\:3.7$ GHz to $\:7.2$ GHz, indicating that the proposed antenna achieves a percent bandwidth (%BW) of 60%.

### Measurement of the gain of the free-standing antenna

**Fig. 11:**
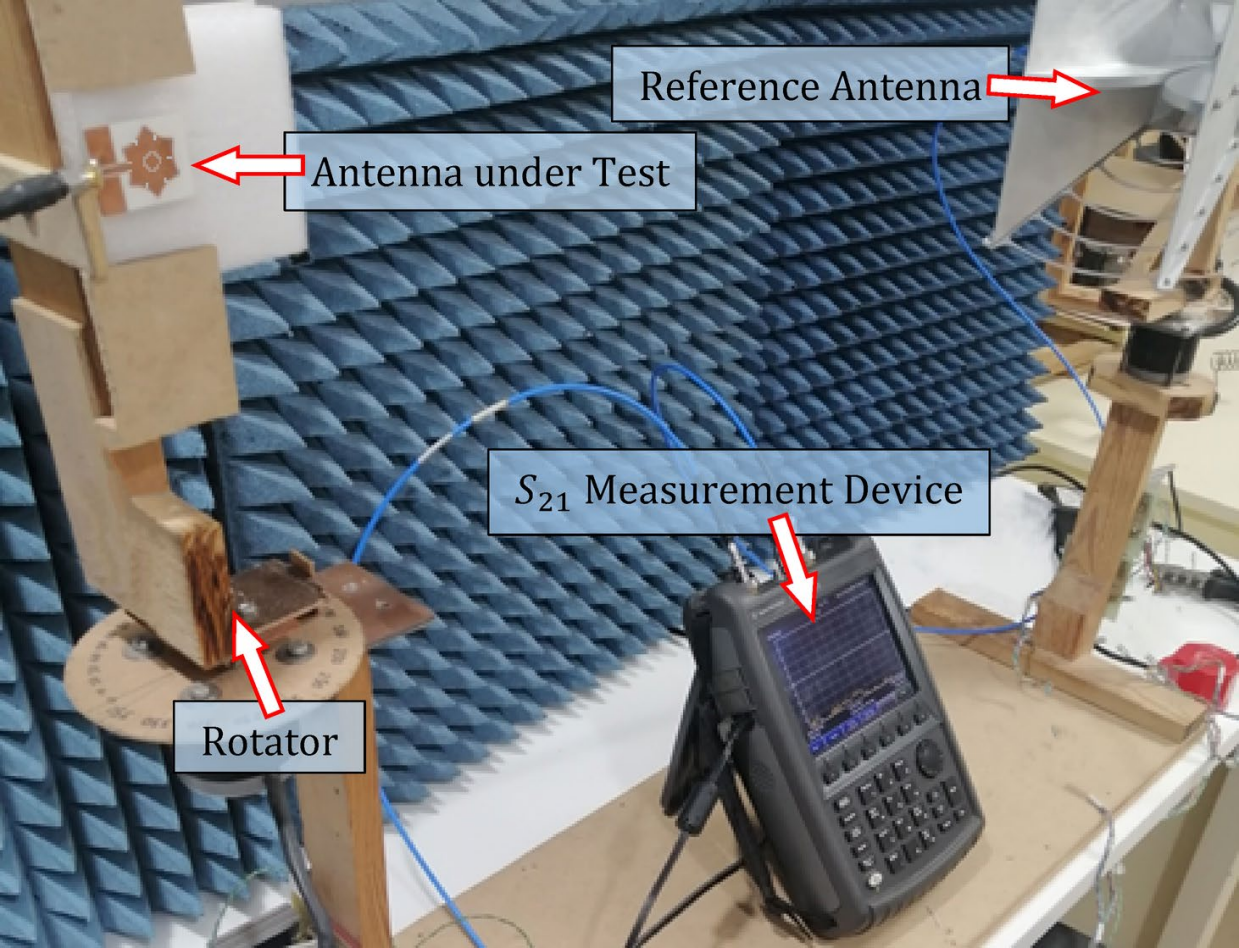
Measurement of the far-field antenna parameters.

The setup depicted in Fig. 11 is utilized to evaluate the antenna’s performance parameters, including gain and radiation patterns, across the frequency range of 2–8 GHz. The Agilent N9918 A VNA is employed to measure the $\:{S}_{21}$​ parameter, with the test antenna and reference antenna connected to port 1 and port 2 of the VNA, respectively. The reference antenna used in this setup is an A-info LB-10,180-SF horn antenna (operating within 10–18 GHz). The test antenna is mounted on a rotating stepper motor to enable 3D radiation pattern measurements. A custom-designed laptop program serves as the central control system, synchronizing the motor’s rotation with data acquisition, managing the measurement process, and displaying the results.

**Fig. 12:**
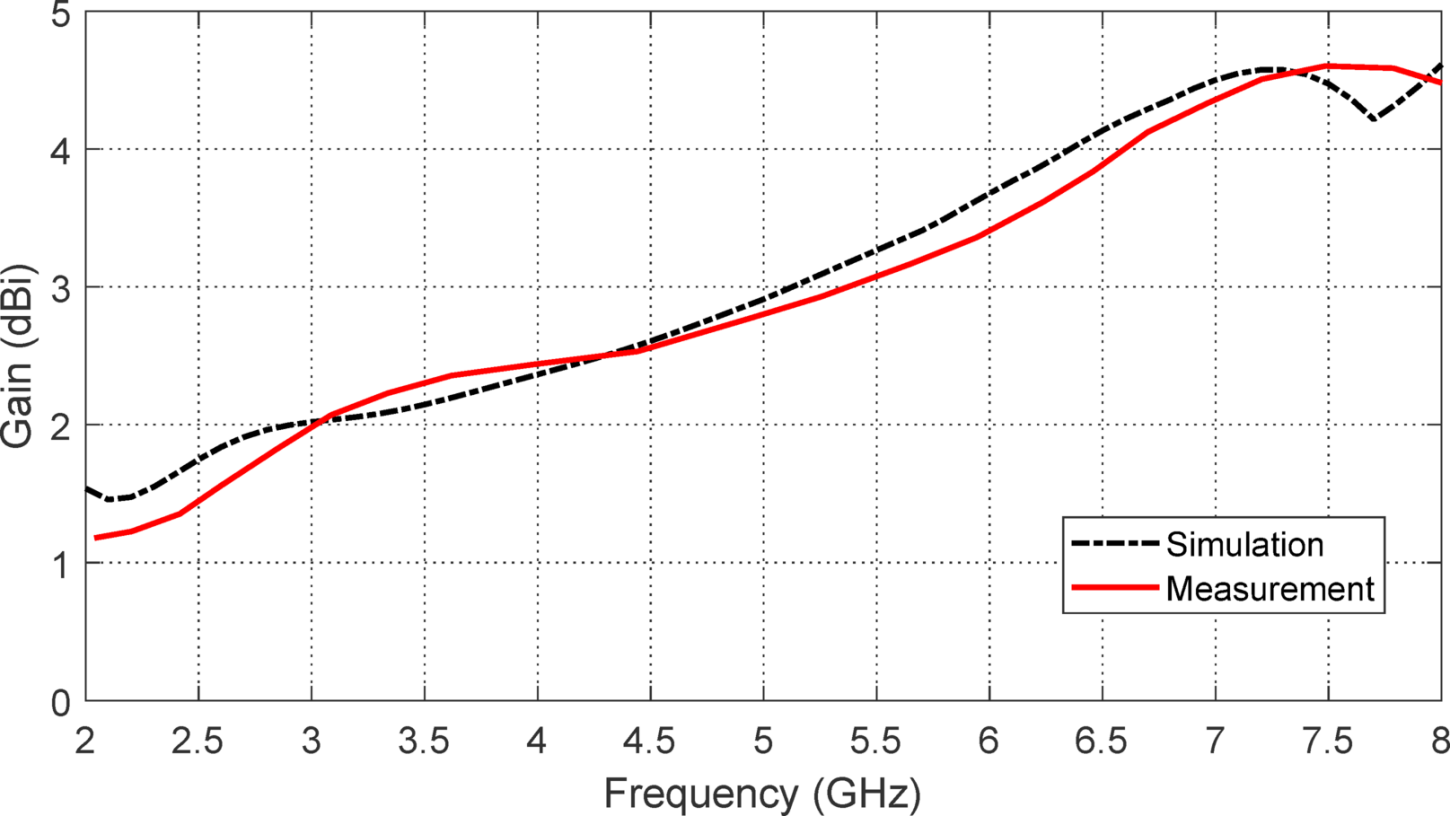
Results of simulation and measurement for the free-standing antenna gain over the frequency range 2–8$2-8$ GHz.

The maximum gain of the free-standing antenna is evaluated both numerically and experimentally as a function of frequency. The results are displayed in Fig. 12, demonstrating a strong correlation between the simulation outcomes and the experimental measurements. Due to the antenna’s omnidirectional nature within the operating frequency range, its maximum gain varies between $\:2$ dBi and $\:4.5$ dBi.

## Radiation characteristics of the free-standing antenna

The radiation characteristics of the proposed wideband antenna in free space, including far-field parameters such as gain, power patterns and efficiency, are presented and analyzed in the following subsections.

### Far-field patterns of the free-standing antenna

**Fig. 13:**
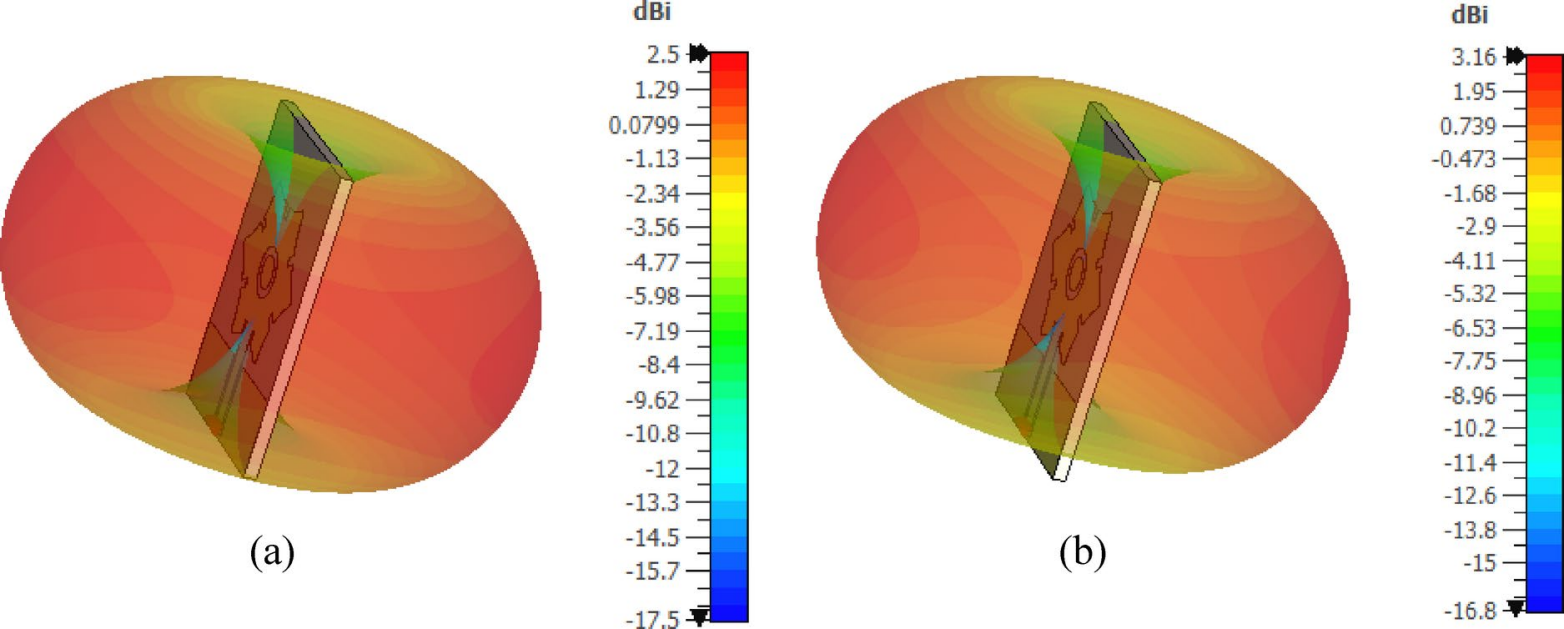
3D gain patterns of the free-standing antenna at (**a**) 4 GHz and (**b**) 5GHz.

**Fig. 14:**
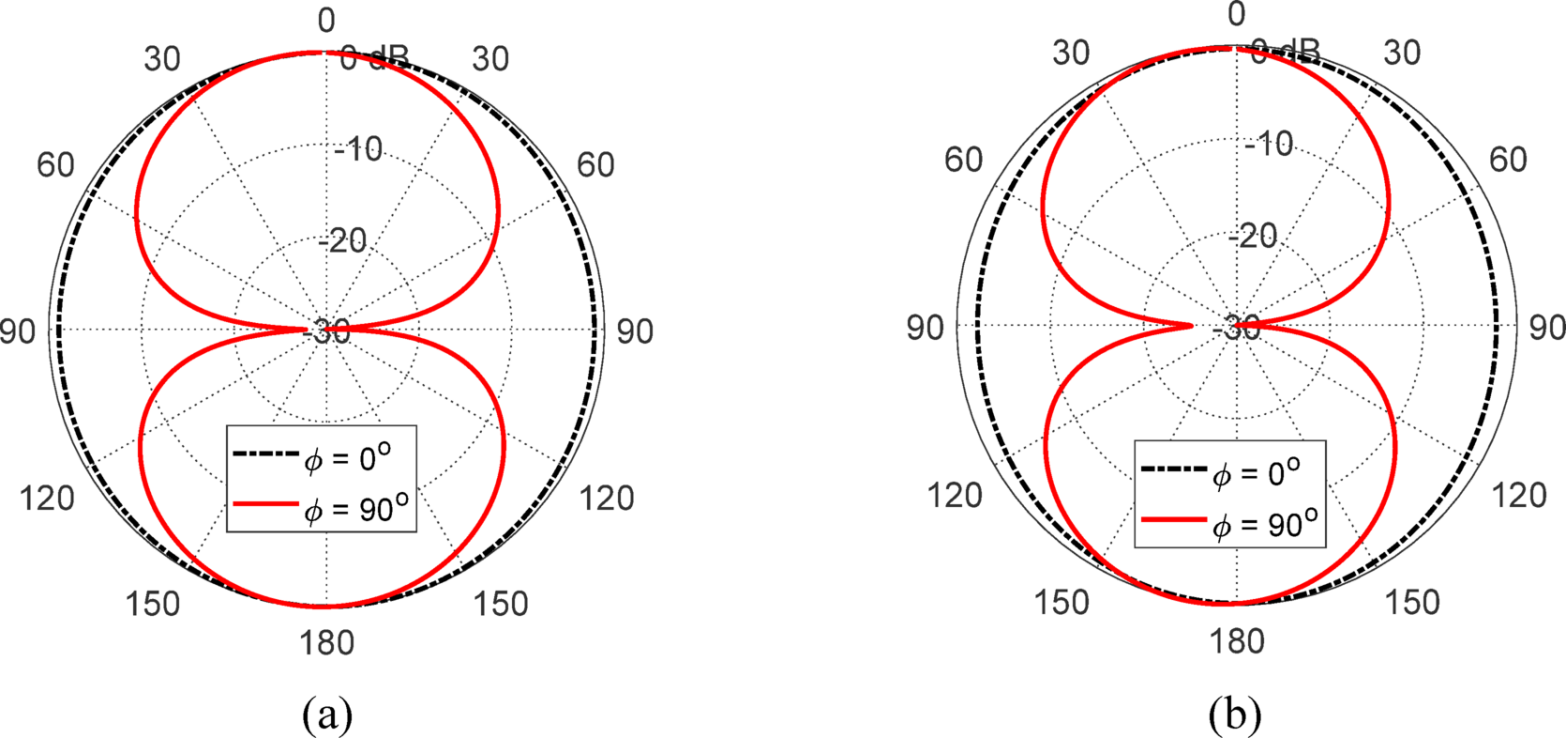
Normalized radiation patterns of the free-standing antenna in the planes *ϕ* = 0° and *ϕ* = 90° at (**a**) 4 GHz and (**b**) 5GHz.

The 3D gain patterns of the free-standing antenna at 4 and 5 GHz are displayed in Fig. 13. The gain pattern is observed to be omnidirectional, with two distinct nulls. The gain values at 4 and $\:5$ GHz are $\:2.5$ dBi and $\:3.1$6 dBi, respectively. Figure 14 presents the 2D normalized far-field patterns of the proposed free-standing star-shaped antenna at $\:4$ and $\:5$ GHz in the elevation planes $\:\varphi\:=0^\circ\:$ and $\:\varphi\:=90^\circ\:$. These patterns reveal that the free-standing antenna exhibits a figure-of-eight radiation pattern, similar to the conventional omnidirectional dipole and monopole antennas.

### Radiation efficiency of the free-standing antenna

**Fig. 15:**
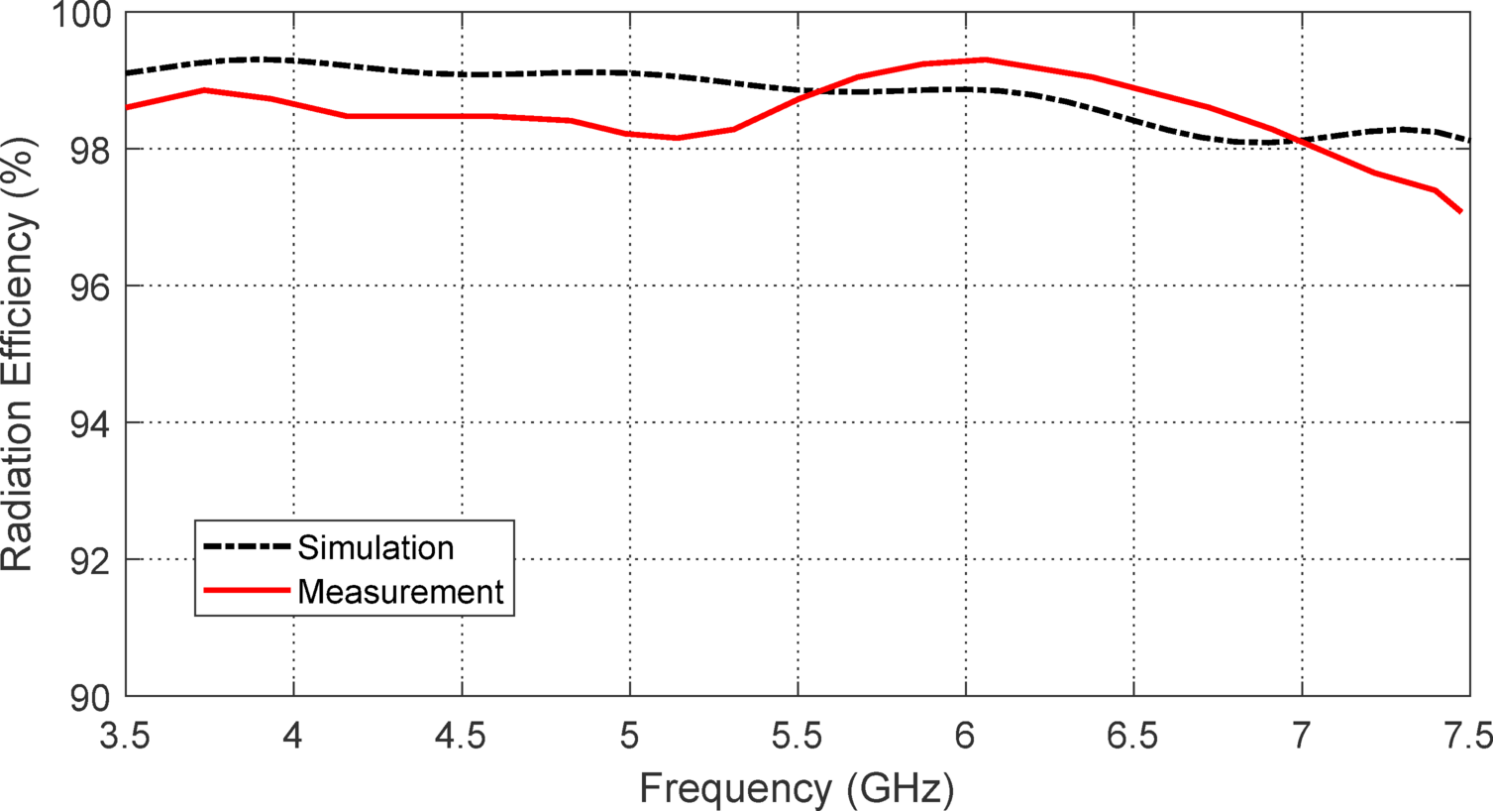
Radiation efficiency of the free-standing printed monopole star-shaped patch antenna at ($3.5-7.5\:\text{G}\text{H}\text{z}$).

**Fig. 16:**
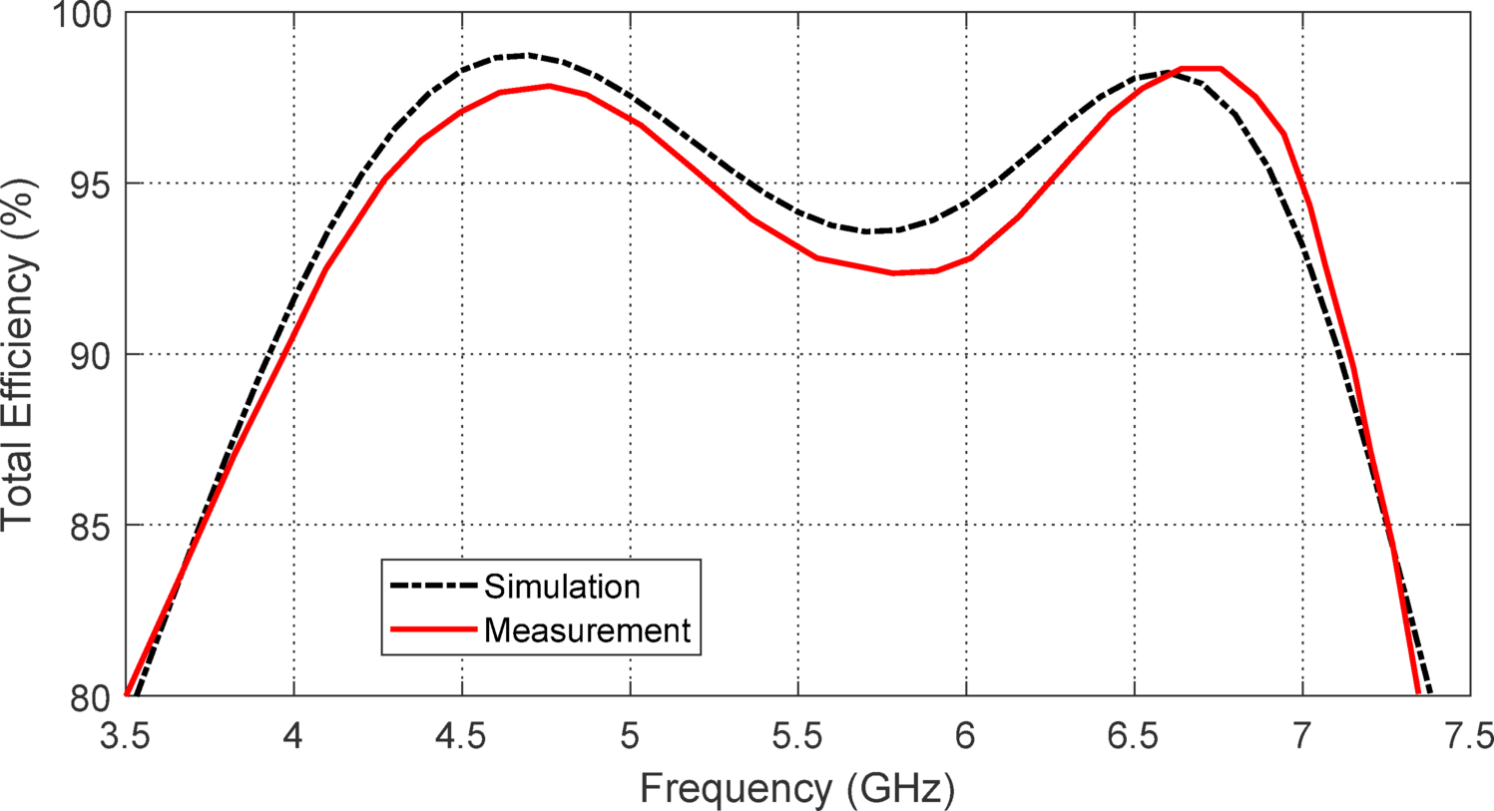
Total efficiency of the free-standing printed monopole star-shaped patch antenna at ($3.5-7.5\:\text{G}\text{H}\text{z}$).

The variation in radiation efficiency of the proposed star antenna across frequencies is illustrated in Fig. 15. The total efficiency is also calculated and plotted in Fig. 16. The radiation efficiency remains above $\:98\%$ throughout the entire operating frequency band. In contrast, the total efficiency stays above $\:85\%$ across the $3.9-7.2$ GHz frequency range. The high efficiency of this antenna can be attributed to its single-sided structure and the CPW feeding, which alleviates field confinement within the substrate, as there is no ground plane present.

## Design of the AMCS for wideband operation

Omnidirectional antennas typically have lower gain compared to directive antennas. However, their gain can be enhanced by placing a metallic surface behind them. When the antenna is positioned at an optimal distance from a properly sized metallic surface, the surface acts as a reflector, causing the backscattered field to add constructively to the radiated field. This increases the antenna’s radiation in the forward direction while reducing radiation toward the rear (toward the reflector). Since the metallic reflector is a perfect electric conductor, the reflected field undergoes a phase shift of approximately 180° relative to the incident field.

If the antenna is too close to the metallic reflector, destructive interference between the forward radiated field and the backscattered field can reduce the overall radiation efficiency. To avoid this, the antenna must be placed sufficiently far from the reflector. However, this increases the size of the radiating structure, including both the antenna and the reflector. One solution to this size issue is replacing the metallic reflector, a perfect electric conducting surface (PECS), with a perfect magnetic conducting surface (PMCS). A PMCS reflects the fields in-phase with the incident waves, leading to constructive interference and enhanced gain, even when the antenna is placed near the PMC surface.

Since a naturally occurring PMCS is difficult to implement, it can be realized as a metasurface using a planar periodic structure, known as an Artificial Magnetic Conducting Surface (AMCS). This section introduces the design of the proposed AMCS and investigates its characteristics through simulations to determine the optimal unit cell dimensions. The frequency band of the resulting AMCS is chosen to be compatible with the wideband two-arm monopole printed antenna discussed in previous sections (Sects. 2 through 4).

### Design of the AMCS unit cell

The AMC surface is designed to exhibit high surface impedance, which corresponds to a reflection coefficient with a phase of $\:0^\circ\:$ at resonance. The effective relative permittivity ($\:{\epsilon\:}_{r}$​) and relative permeability ($\:{\mu\:}_{r}$​) must result in a wave impedance ($\:Z=\sqrt{{\mu\:}_{r}/{\epsilon\:}_{r}}$).​ For AMCS, the effective relative permittivity is typically engineered to be low to moderate and can vary depending on the capacitance introduced by the AMCS unit cell design. Thus, $\:{\epsilon\:}_{r}$ is usually close to 1 (air-like behavior) or slightly higher, reflecting the structure’s ability to store electric energy. The effective relative permeability is engineered to be moderately high, often greater than 1, to mimic the behavior of a magnetic conductor. The inductive effects in the AMCS unit cells (such as loop or meander structures) contribute to increasing the effective ​$\:{\mu\:}_{r}$. Typical ranges for AMC operation at or near resonance are: $\:1\le\:{\epsilon\:}_{r}\le\:5$ and $\:1<{\mu\:}_{r}\le\:10$. The higher $\:{\mu\:}_{r}$ helps in achieving the AMC condition with a $\:0^\circ\:$ reflection phase and suppressing surface waves.

Consider an AMCS designed for antenna applications, $\:{\epsilon\:}_{r}\approx\:2$ represents low-to-moderate electric field storage capacity whereas, $\:{\mu\:}_{r}\approx\:6$ mimics strong magnetic field interactions due to the inductive nature of the unit cells. Such a design could enable the AMC to act as a reflector for enhancing antenna gain while maintaining the desired resonance properties. By carefully controlling the geometry and materials of the AMCS unit cells, the relative permittivity and permeability can be tuned to meet the specific requirements of the application.

**Fig. 17:**
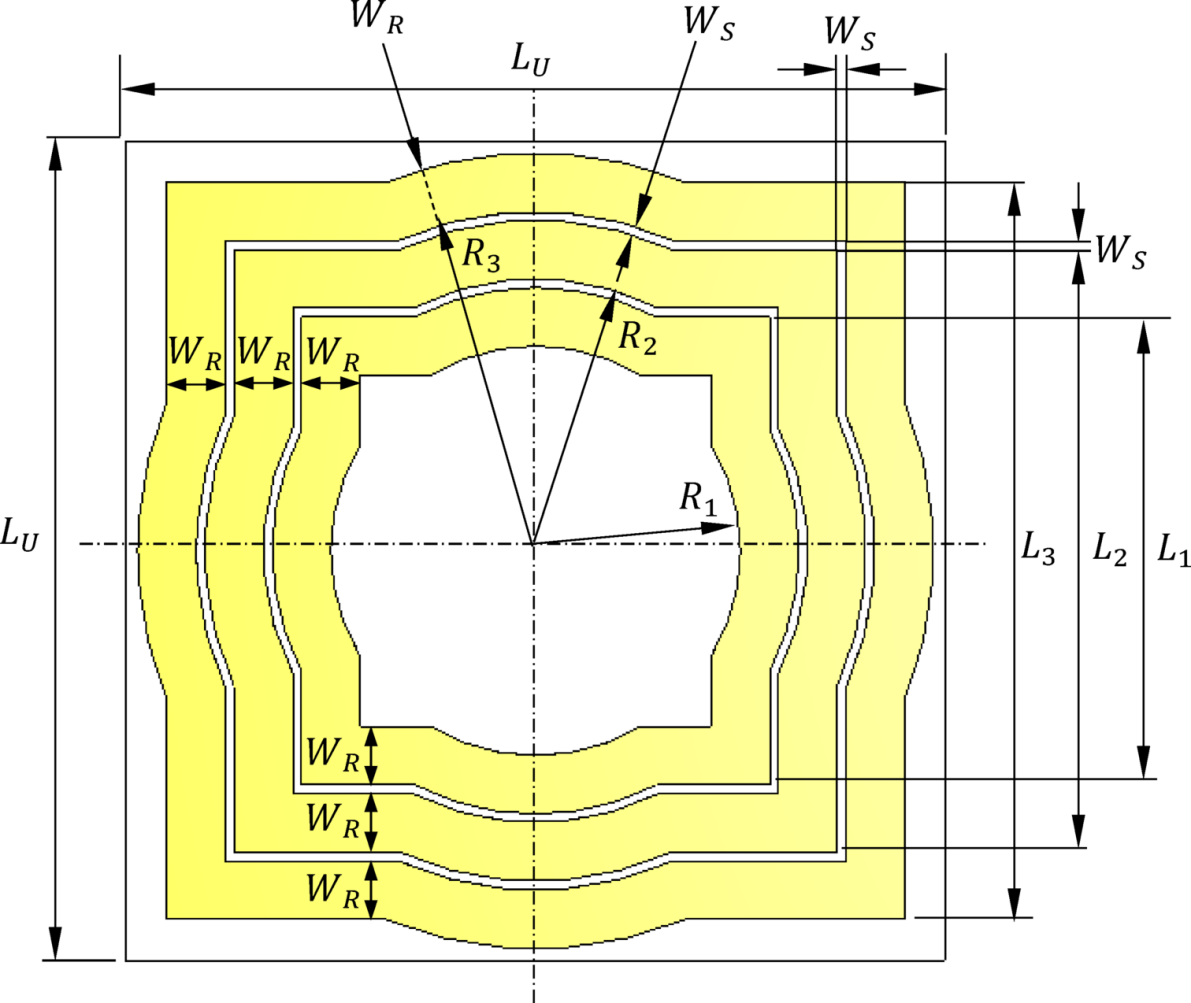
Geometry of the unit cell of the AMCS proposed to enhance the antenna gain.

The design of the proposed AMCS unit cell is illustrated in Fig. 17. The unit cell consists of three concentric metallic frames, each formed by combining a square and a circular frame, as depicted in the figure. These frames are printed on the top surface of a dielectric substrate made of Roger’s RO4003 C, with the same material and thickness as that used for the wideband monopole two-arm patch antenna described in Sect. Design of the Wideband Free-Standing Antenna.

**Fig. 18:**
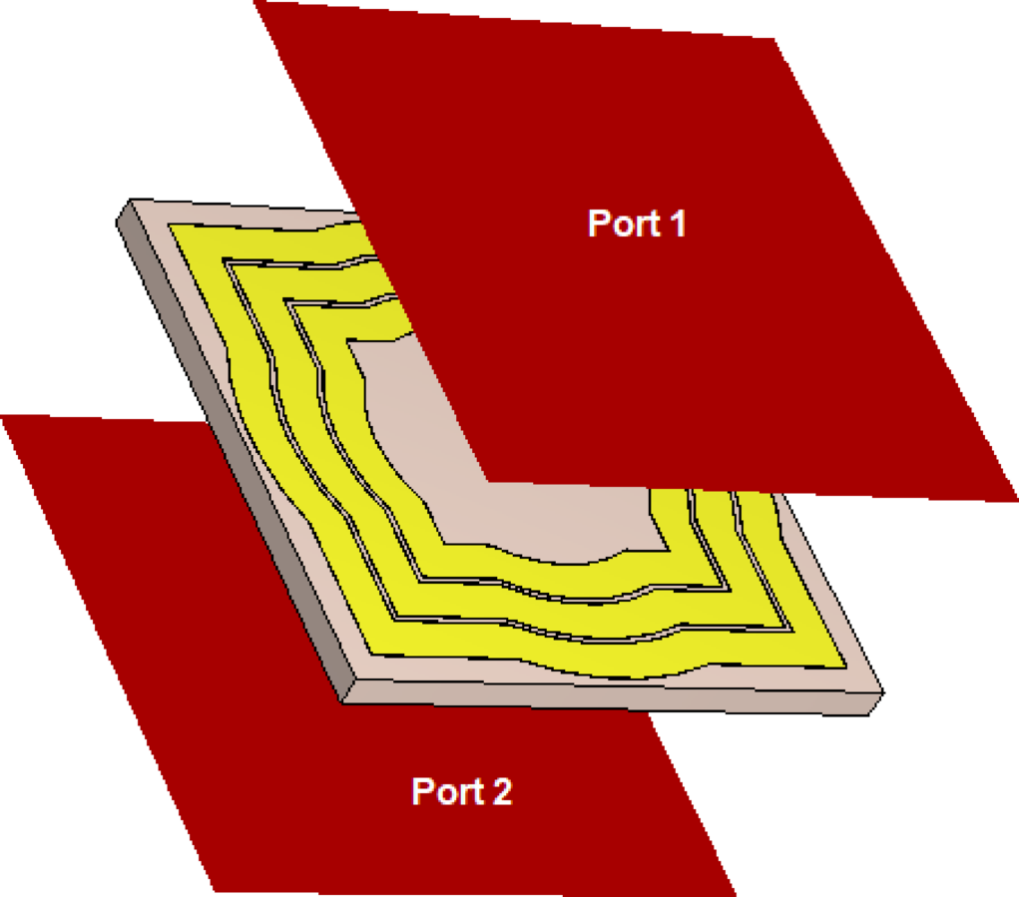
Simulation of the AMCS unit cell.

To achieve a wideband AMCS, the bottom side of the dielectric substrate is left bare, with the copper layer removed. This results in a single-sided printed AMCS structure. The unit cell design was optimized using the CST simulator to maximize the bandwidth of the AMCS. The simulation model for the unit cell is shown in Fig. 11. The design features a printed pattern on the top side of the dielectric substrate without a solid ground plane on the bottom side. Therefore, two ports (Port 1 and Port 2) are used for the simulation, as indicated in Fig. 18. The optimized dimensions of the AMCS unit cell are detailed in Table 2.

**Table 2:** Dimensions of the proposed AMCS unit cell whose geometry is shown in Fig. 17.

Parameter	$\:{R}_{1}$	$\:{R}_{2}$	$\:{R}_{3}$	$\:{L}_{1}$	$\:{L}_{2}$	$\:{L}_{3}$	$\:{W}_{R}$	$\:{W}_{S}$	$\:{L}_{U}$
Value (mm)	$\:10$	$\:12.3$	$\:14.6$	$\:18$	$\:22.6$	$\:27.2$	$\:2.0$	$\:0.3$	$\:30$

### Reflection and transmission properties of the AMCS

**Fig. 19:**
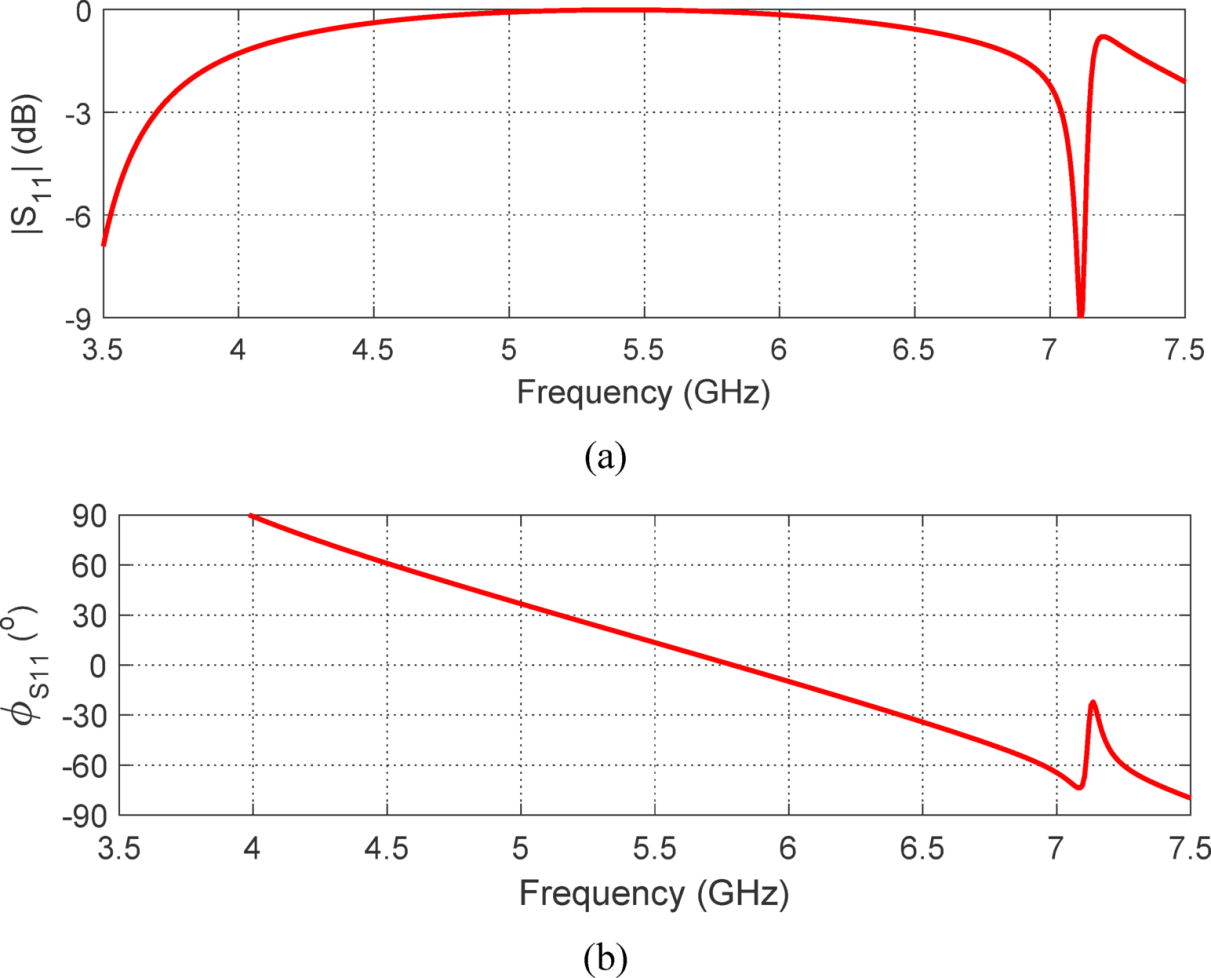
Frequency dependence of $\:\left|{S}_{11}\right|$ considering the unit cell excitation model shown in Fig. 11.

**Fig. 20:**
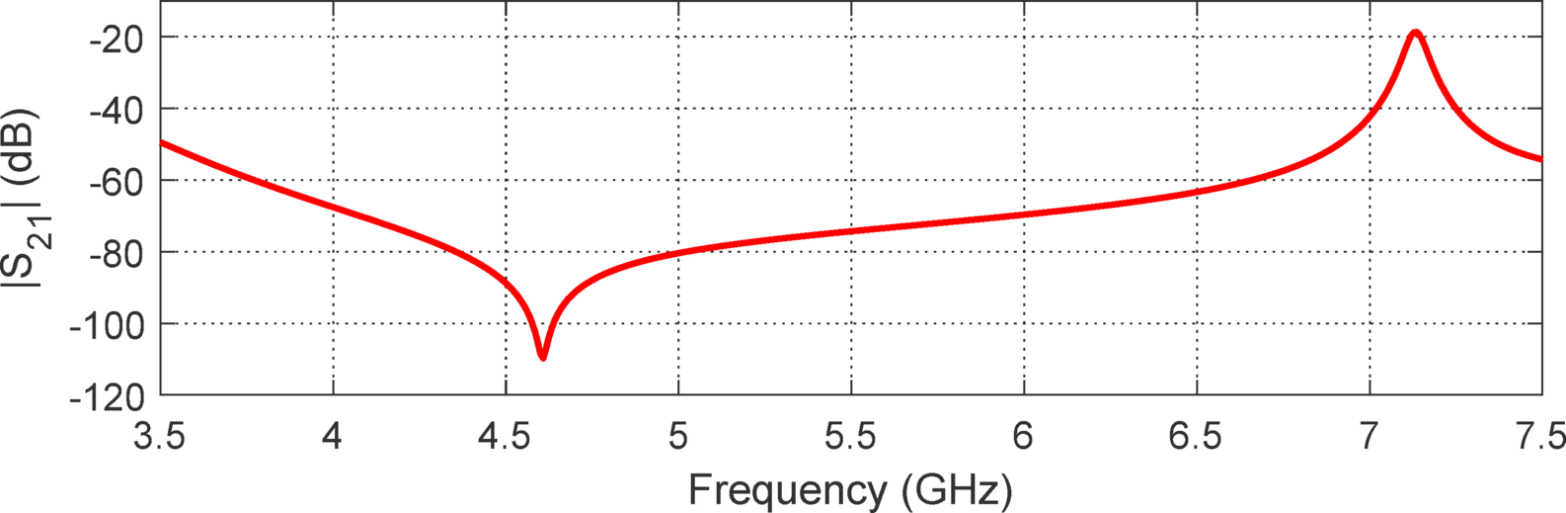
Frequency dependence of $\:\left|{S}_{21}\right|$ considering the unit cell excitation model shown in Fig. 11.

The results for $\:{S}_{11}$ and $\:{S}_{21}$​ as a function of frequency are shown in Figs. 19 and 20, respectively. As seen in Fig. 19a, the magnitude of $\:\left|{S}_{11}\right|$ meets the condition $\:\left|{S}_{11}\right|\:>-3\:\text{d}\text{B}$ within the frequency range of $\:3.5$ to 7.2 GHz. Additionally, the phase of ​$\:{S}_{11}$, shown in Fig. 19b, satisfies the condition $\:-90^\circ\:\le\:{{\Phi\:}}_{{S}_{11}}\le\:90^\circ\:$ over the frequency range of $\:3.7$ to $\:7.3$ GHz. Here, where $\:{{\Phi\:}}_{{S}_{11}}$ represents the phase angle of $\:{S}_{11}$. Therefore, the operating frequency band of the proposed AMCS is $\:3.7$ to $\:7.2\:$GHz, corresponding to a $\:68\%$ bandwidth. The magnitude of the transmission coefficient, $\:\left|{S}_{21}\right|$, shown in Fig. 23, remains below $\:-60\:$dB across the AMCS operating range ($\:3.5$ to $\:7.2$ GHz). This indicates that the surface effectively functions as a reflector, enhancing the gain of wideband antennas.

### AMCS of finite dimensions

**Fig. 21:**
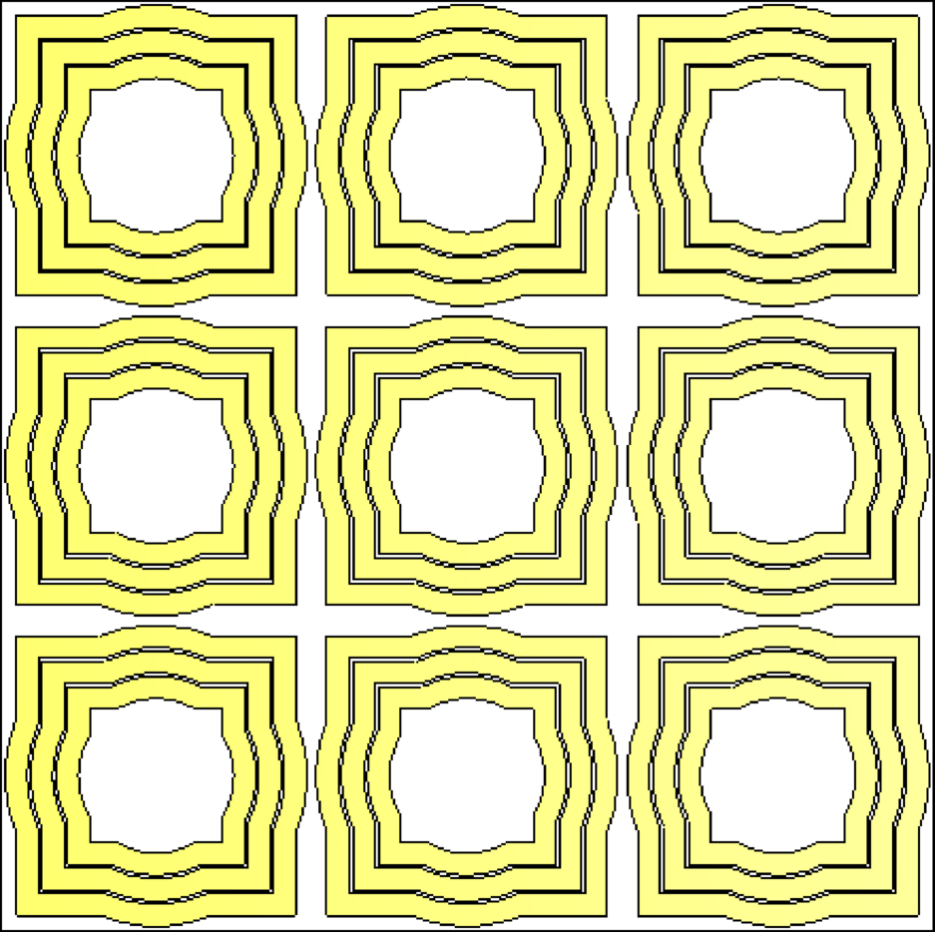
AMCS constructed of $\:3\times\:3$ unit cells.

**Fig. 22:**
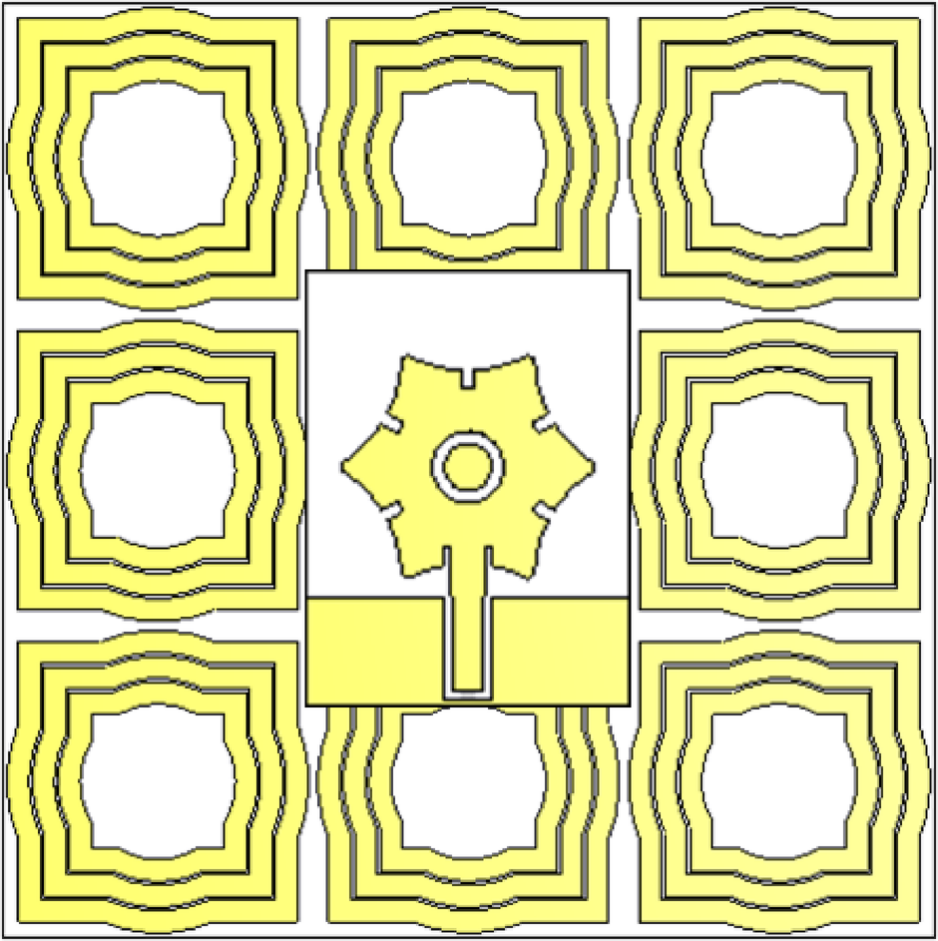
The monopole star-shaped patch antenna is placed at a height $\:H=23\:\text{m}\text{m}$ above an AMCS of $\:3\times\:3$ unit cells.

The numerical analysis conducted in Sect. 6.2 focuses on a unit cell of the proposed AMCS under periodic boundary conditions along the side boundaries of the solution region. This implies that the investigations consider an AMCS that extends infinitely. However, for practical applications, particularly in antenna gain enhancement, it is advisable to reduce the size of the reflecting surface. Therefore, this section examines the performance of a finite AMCS with the smallest possible number of cells while still achieving the desired gain enhancement for the antenna. The performance of the complete antenna structure, supported by the AMCS, is evaluated. Additionally, to minimize the profile of the resulting structure, it is recommended to decrease the distance between the antenna and the AMCS reflector, provided the necessary gain enhancement is maintained. Figure 21 illustrates the CST model of an AMC surface consisting of a $\:3\times\:3$ unit cells configuration. Figure 22 shows the wideband monopole planar antenna (described in Sect. 2) while being placed at a distance $\:H$ over the $\:3\times\:3$-cell AMCS described above

### Parametric study for the antenna over the AMCS

This section examines the impact of various design parameters of the AMCS unit cell on the performance of the star-shaped monopole antenna when positioned above the AMCS. Additionally, the influence of the antenna’s height relative to the AMCS is analyzed, presented, and discussed.

#### Effects of the AMCS design parameters on the antenna performance

**Fig. 23:**
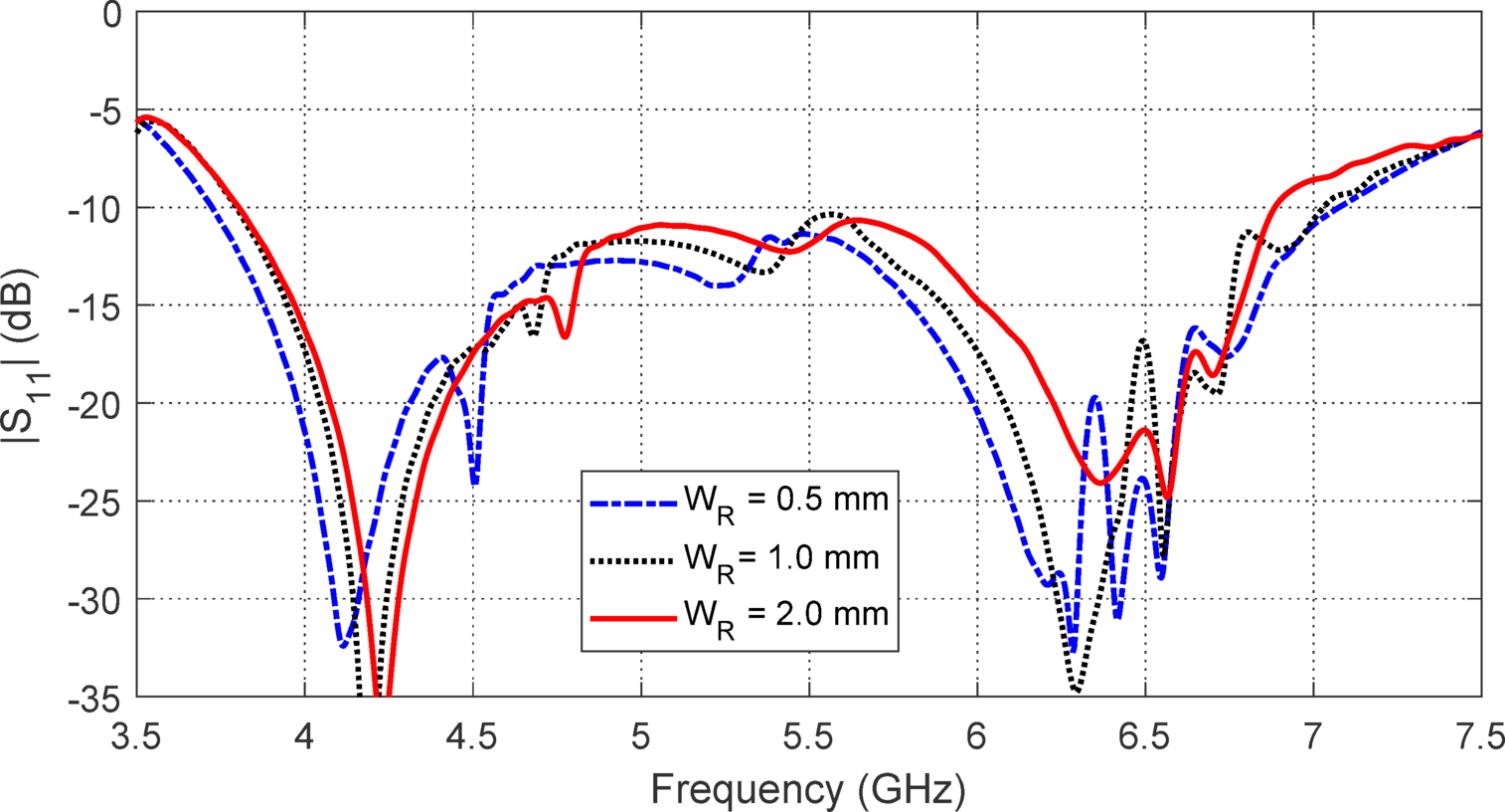
Effect of changing the width $\:{W}_{R}$ of the strip ring of the AMCS unit cell on the frequency dependence of $\:\left|{S}_{11}\right|$ of the antenna over the AMCS.

**Fig. 24:**
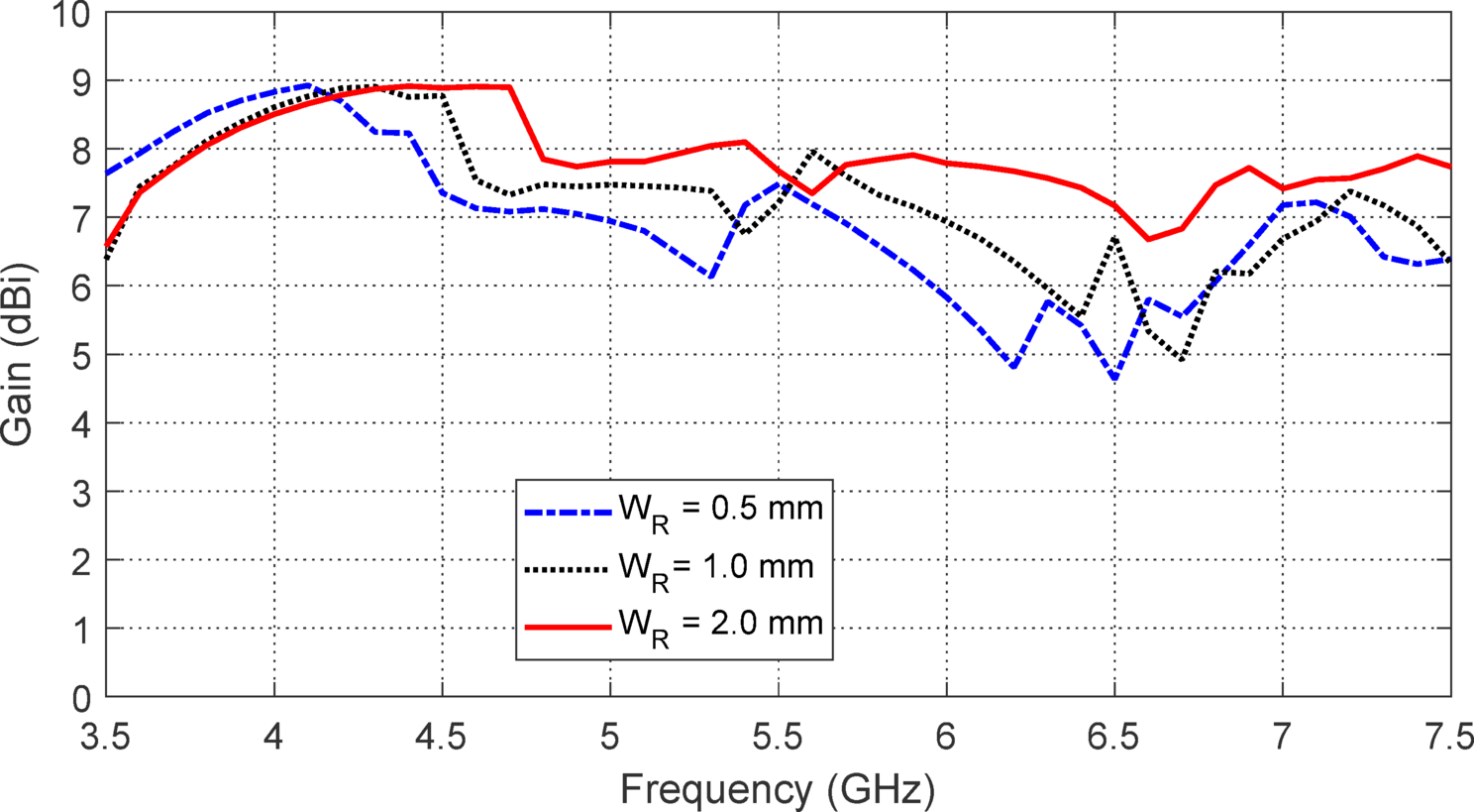
Effect of changing the width $\:{W}_{R}$ of the strip ring of the AMCS unit cell on the frequency dependence of maximum gain produced by the antenna over the AMCS.

The effect of varying the strip ring width WRW_RWR​ in the AMCS unit cell on the frequency dependence of $\:\left|{S}_{11}\right|$ and the antenna gain is shown in Figs. 23 and 24, respectively. It is observed that $\:{W}_{R}=0.5\:\text{m}\text{m}$ results in the widest frequency band, as depicted in Fig. 24. However, the highest gain and the most stable maximum gain across the operational frequency range are achieved when $\:{W}_{R}=2.0\:\text{m}\text{m}$ as shown in the same figure. Therefore, $\:{W}_{R}=2.0\:\text{m}\text{m}$ is chosen as it provides the highest and most stable gain, with a satisfactory bandwidth.

**Fig. 25:**
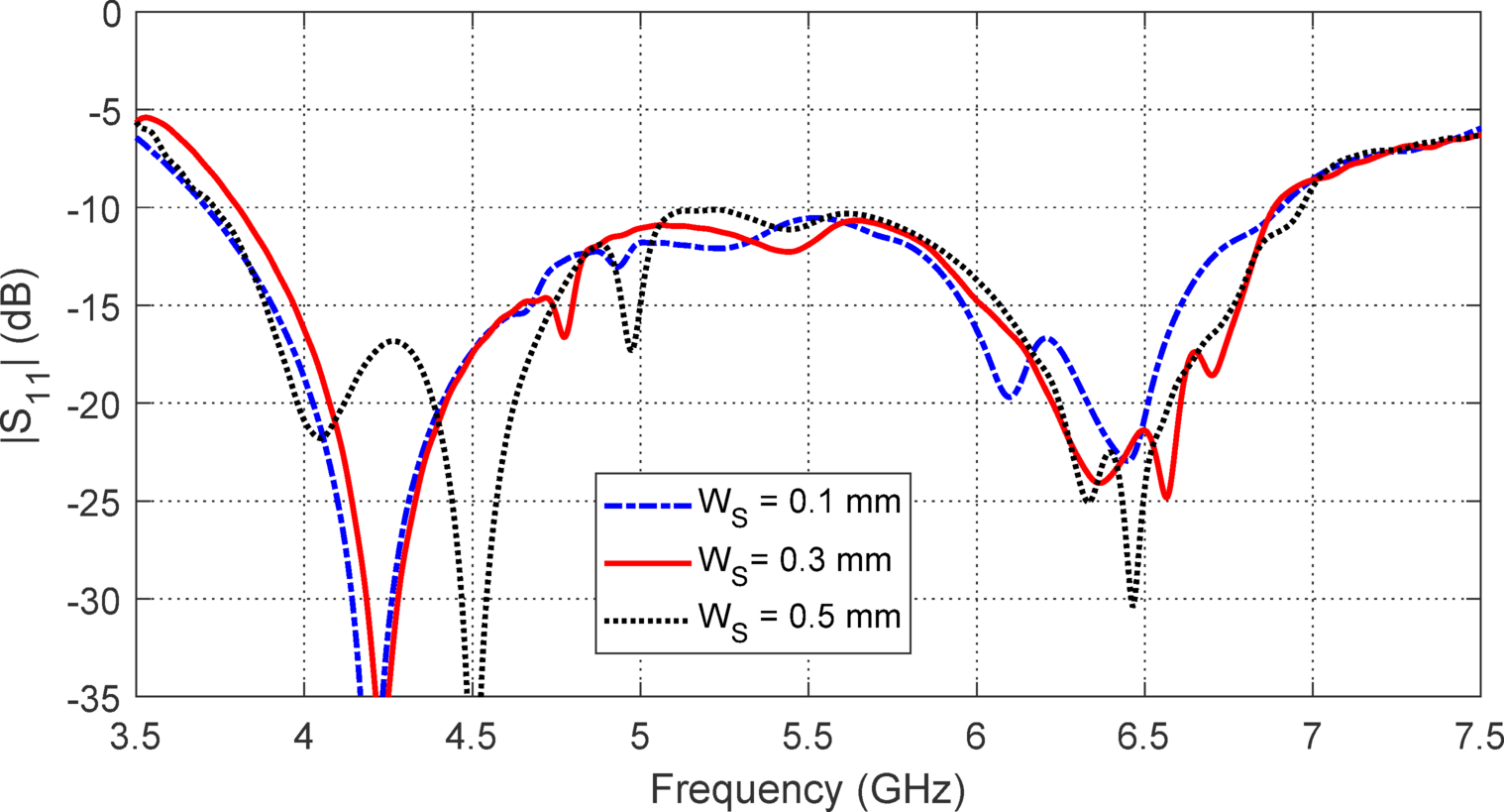
The effect of changing the separation $\:{W}_{S}$ between the strip rings of the AMCS unit cell on the frequency dependencies of $\:\left|{S}_{11}\right|$ of the antenna over the AMCS.

**Fig. 26:**
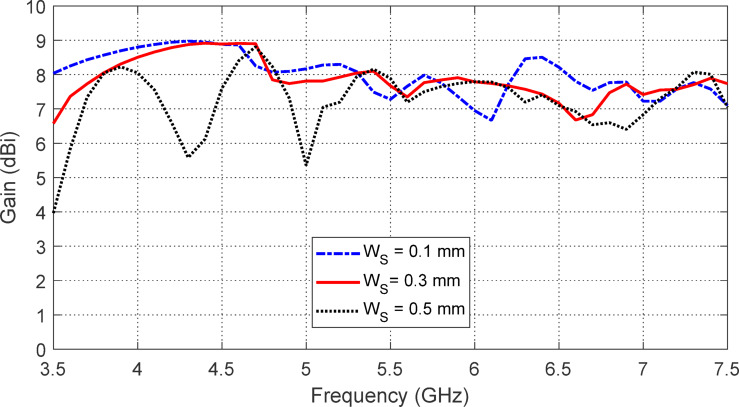
The effect of changing the separation $\:{W}_{S}$ between the strip rings of the AMCS unit cell on the maximum gain produced by the antenna over the AMCS.

The effect of varying the separation $\:{W}_{S}$ between the strip rings of the AMCS unit cell on the frequency dependence of $\:\left|{S}_{11}\right|$ and the antenna gain is shown in Figs. 25 and 26, respectively. It is observed that $\:{W}_{S}=0.5\:\text{m}\text{m}$ results in the widest frequency band, as shown in Fig. 25. However, the highest gain and the most stable maximum gain across the operational frequency range are achieved when $\:{W}_{S}=0.3\:\text{m}\text{m}$, as shown in Fig. 26. Therefore, $\:{W}_{S}=0.3\:\text{m}\text{m}$ is chosen, as it provides the most stable gain with a satisfactory bandwidth.

#### Effects of the antenna height over the AMCS

**Fig. 27:**
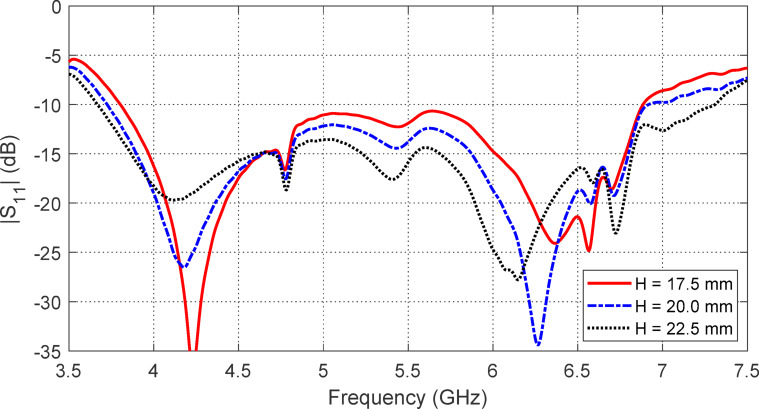
The effect of changing the antenna height, $\:H$, over the AMCS on the frequency dependence of $\:\left|{S}_{11}\right|$.

**Fig. 28:**
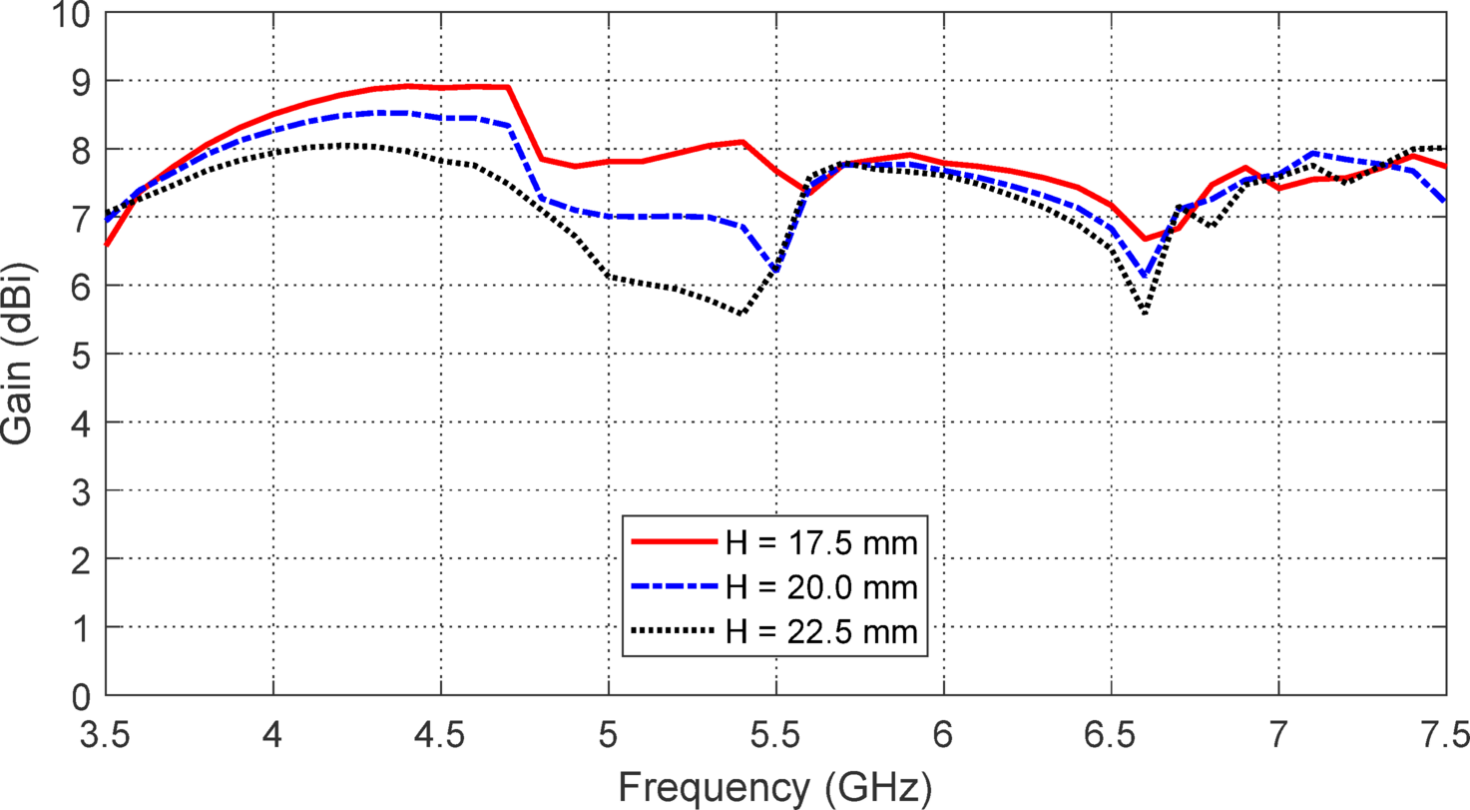
The effect of changing the antenna height, $\:H$, over the AMCS on the frequency dependence of the maximum gain.

The frequency behaviors of $\:\left|{S}_{11}\right|$ and the maximum gain produced by the antenna are influenced by the antenna height $\:H$ above the AMCS, as shown in Figs. 27 and 28, respectively. The impedance matching frequency band is extended to $3.65-7.35$ GHz ($\:66\%$) when the antenna is placed at a height of $\:H=22.5\:\text{m}\text{m}$ above the AMCS, compared to the free-standing antenna’s frequency band of $3.9-7.2$ GHz ($\:60\%$). This improvement is attributed to the coupling effect between the antenna and the AMCS. However, the highest gain and the best stability of the gain across the frequency range are achieved when the antenna is positioned at a height of$\:\:H=17.5\:\text{m}\text{m}$ above the AMCS, as shown in Fig. 28. Therefore, $\:H=17.5\:\text{m}\text{m}$ is selected, as it provides the highest and most stable gain over the entire frequency band, along with a satisfactory impedance matching bandwidth.

### Radiation characteristics of the antenna over the AMCS

**Fig. 29:**
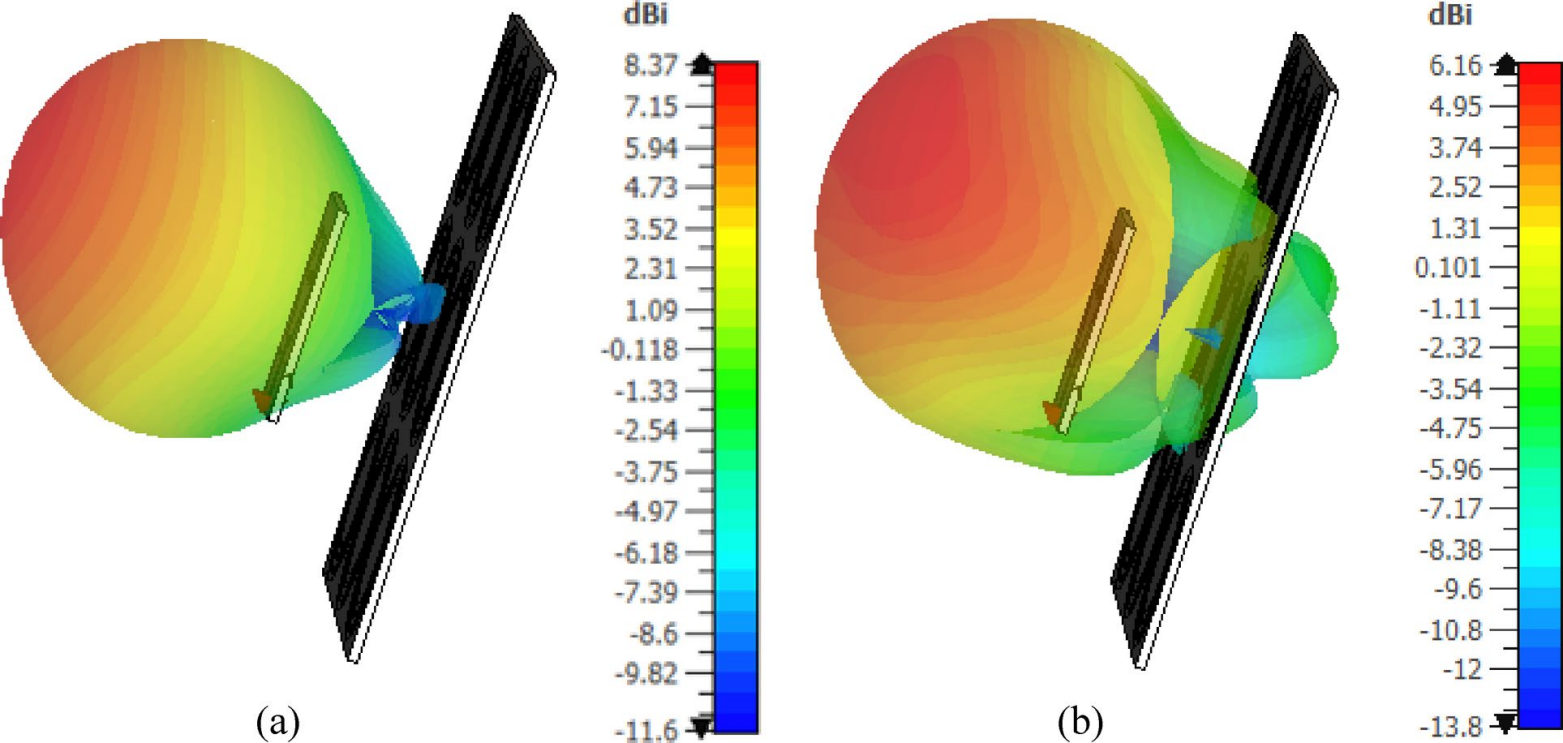
3D gain patterns of the proposed wideband antenna when placed at a height of $\:17.5\:\text{m}\text{m}$ above the proposed AMC surface at (a) $\:4\:\text{G}\text{H}\text{z}$, and (b) $\:5\:\text{G}\text{H}\text{z}$.

**Fig. 30:**
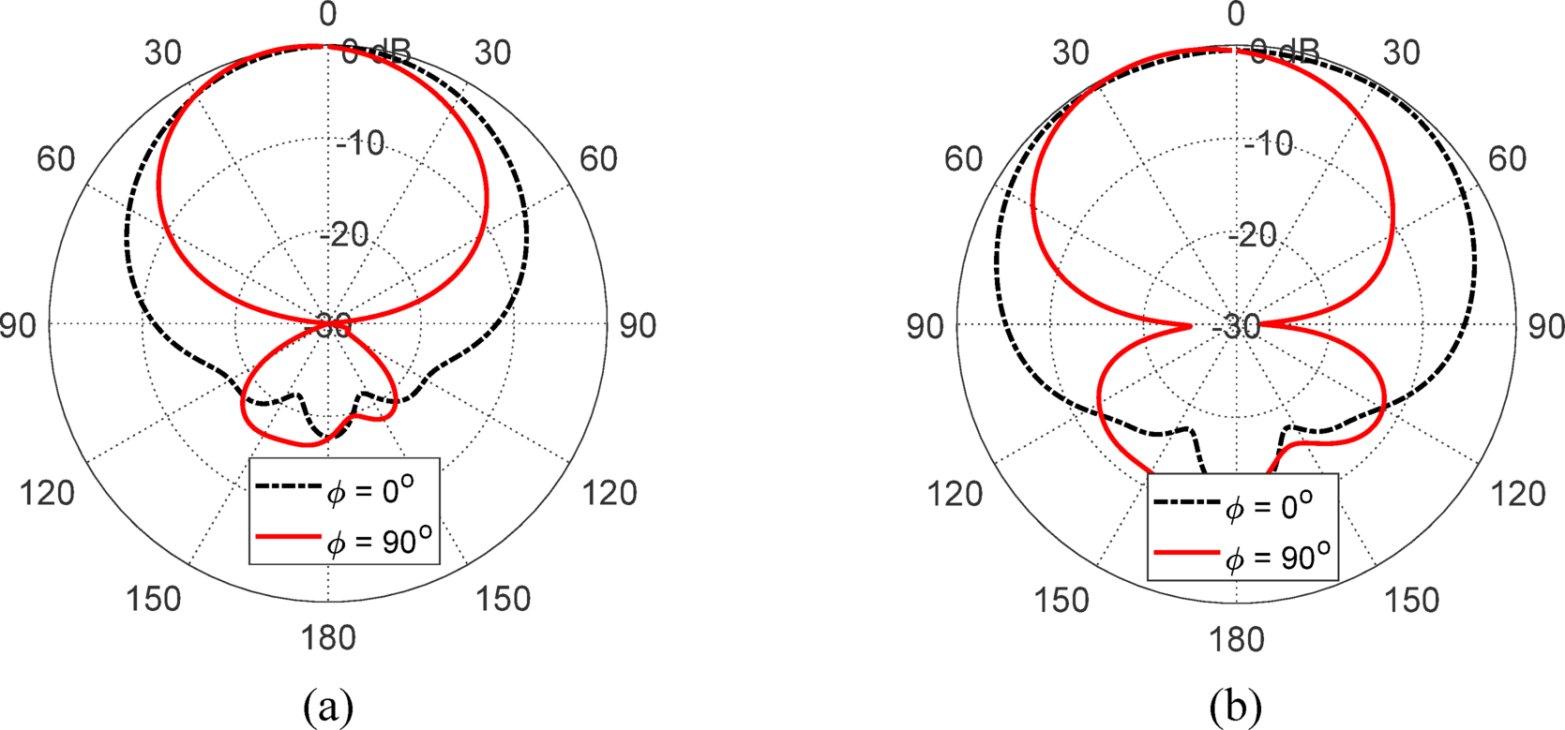
Radiation patterns of the antenna when placed at a height of 17.5 mm over the proposed AMCS at (a) 4 GHz, and (b) 5 GHz.

The 3D gain patterns at 4 and 5 GHz when the finite AMC surface (described in Sect. 6.3) is placed 17.5 mm below the antenna is presented in Fig. 29. The gain pattern is observed to be more directive, with enhanced forward radiation and reduced backward radiation compared to the free-standing antenna (Fig. 13). The addition of the AMCS below the antenna increases the maximum gain from $\:2.5$ dBi to $\:8.37$ dBi at $\:4$ GHz and from $\:3.16$ dBi to $\:6.16$ dBi at $\:5$ GHz. Figure 30 shows the corresponding 2D normalized radiation patterns at $\:4$ and $\:5\:$GHz in the elevation planes $\:\varphi\:=0^\circ\:$ and $\:\varphi\:=90^\circ\:$, demonstrating that the antenna backed by the AMC surface has a directive radiation pattern with a high front-to-back ratio.

**Fig. 31:**
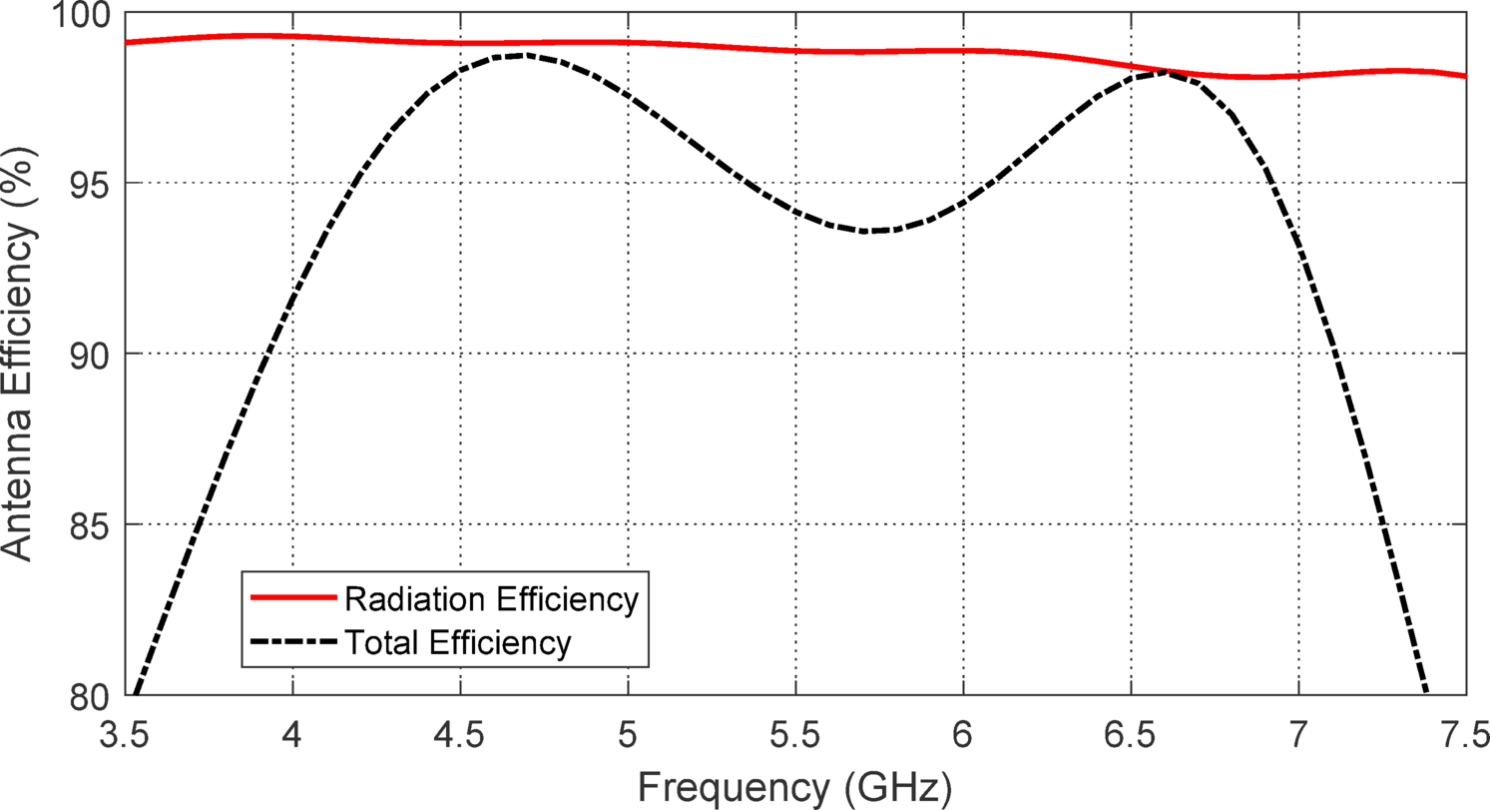
Radiation and total efficiencies of the propose antenna when placed at a height of $\:17.5\:\text{m}\text{m}$ over the AMCS.

Figure 31 illustrates the variation in radiation efficiency for the proposed monopole antenna placed over the designed AMCS. The total efficiency is also plotted in the same figure. It is evident that the radiation efficiency remains above $\:98\%$ across all frequencies of impedance matching, with the total efficiency remaining higher than $\:85\%$ over the frequency band of 3.7–7.2 GHz, compared to the free-standing antenna efficiencies shown in Figs. 15 and 16.

**Fig. 32:**
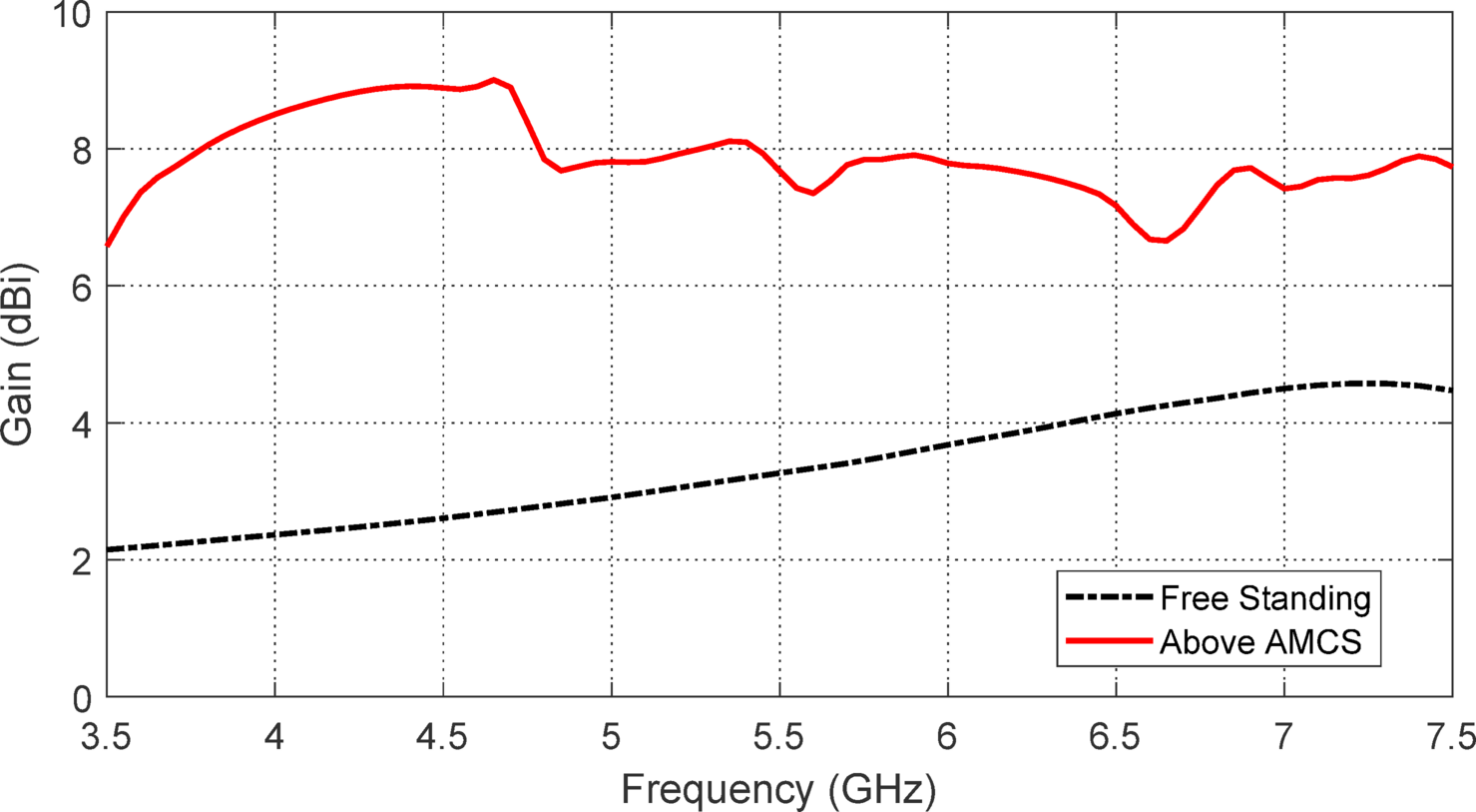
Enhancement of the antenna gain when placed at a height of $\:17.5\:\text{m}\text{m}$ over the AMCS compared with the free-standing antenna gain.

Figure 32 shows the variation of the maximum gain of the proposed antenna with frequency when placed over the designed AMCS, and compares it to the maximum gain of the free-standing monopole antenna. As seen in Fig. 37, the gain is enhanced across the entire operating frequency band ($3.7-7.2$ GHz).

## Fabrication and measurement of the antenna over the AMC surface

This section presents the experimental work conducted to practically evaluate the performance of the proposed wideband antenna when supported by the proposed AMC surface.

### Fabrication of the AMC surface

**Fig. 33:**
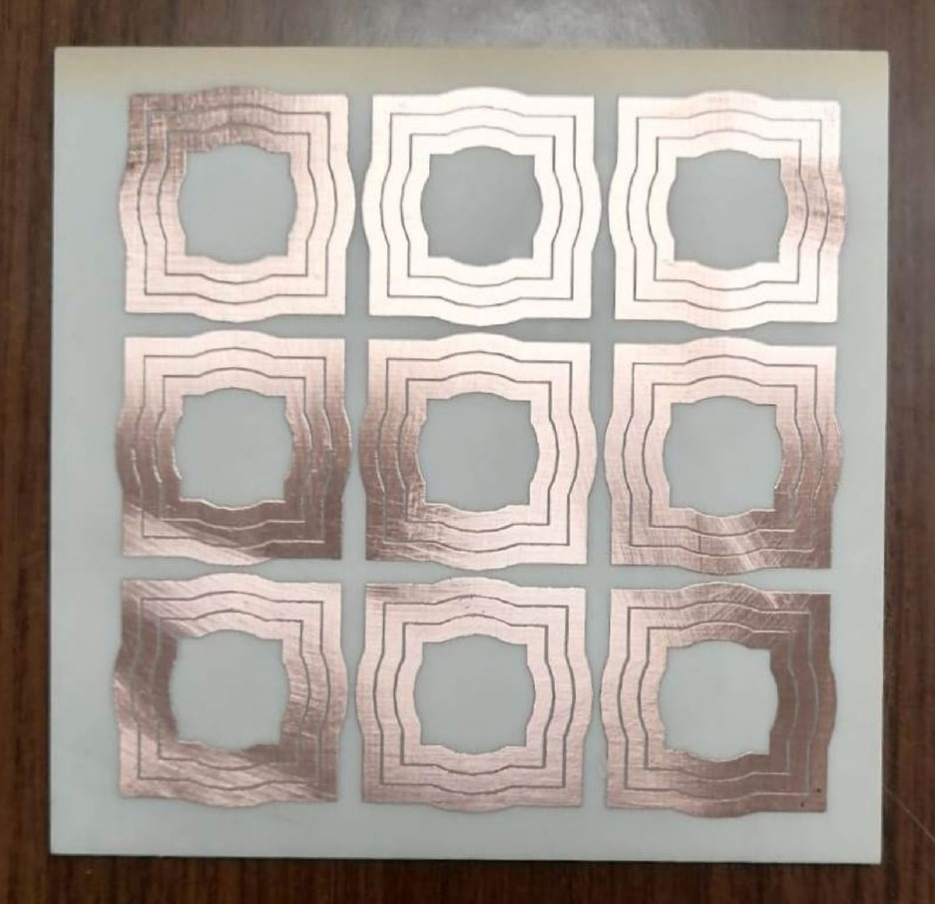
Fabricated prototype of AMCS of $\:3\times\:3$ cells.

An AMCS consisting of 3 × 3 unit cells (as detailed in Sect. 6.3) is fabricated on a single-sided substrate made of Rogers’ RQ4003 C material with a thickness of $\:h=1.52\:\text{m}\text{m}$. This is the same substrate used for fabricating the wideband monopole patch antenna, as described in Sect. 4.1. The fabrication process is carried out through lithography on one side of the substrate, leaving the other side blank. The fabricated AMCS is shown in Fig. 33.

### Placing the antenna over the AMCS

**Fig. 34:**
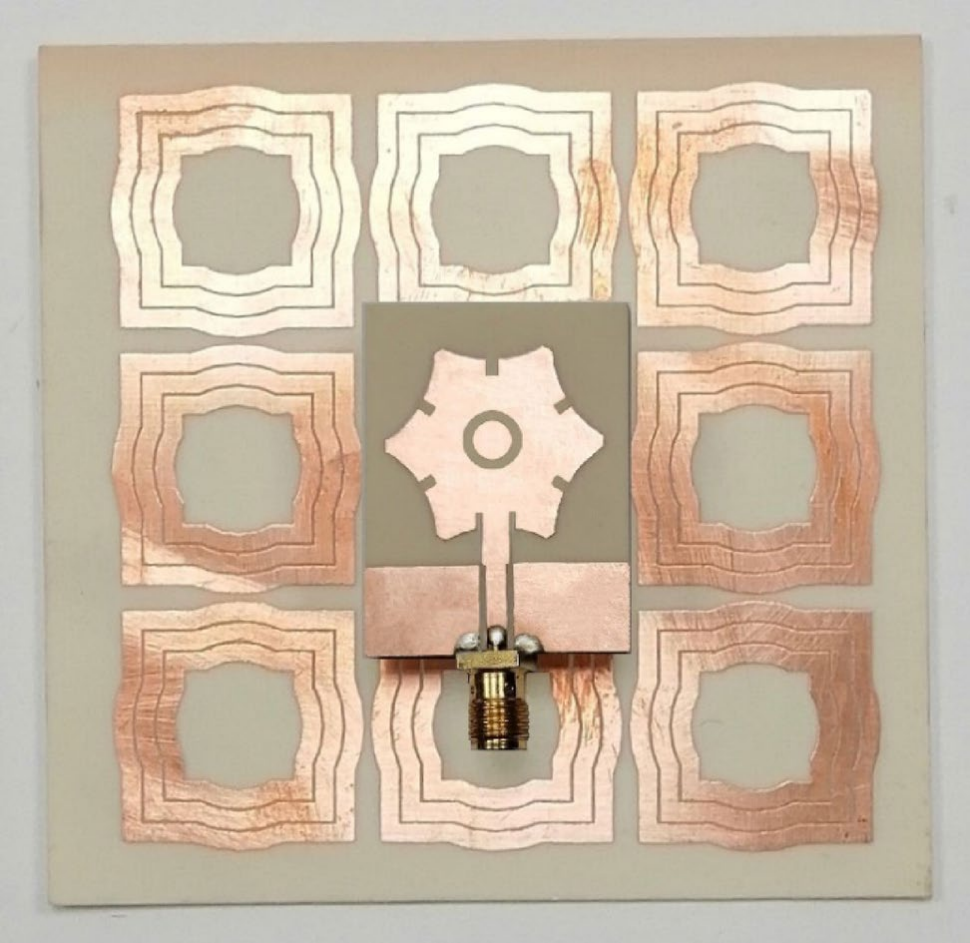
The star-shaped antenna placed at a height of $\:H=17.5\:\text{m}\text{m}$ above the AMC metasurface using a separating block of foam.

The fabricated AMCS is positioned at a distance of $\:H=17.5\:\text{m}\text{m}$ behind the wideband monopole patch antenna, using a foam block of the same thickness, which is securely inserted into the gap between the antenna and the AMCS, as shown in Fig. 34. The foam block serves as a spacer, maintaining the required distance between the antenna and the AMCS. The foam material has a relative permittivity of$\:{\epsilon\:}_{r}=1.01$ and a loss tangent of $\:\text{tan}\delta\:\approx\:0$, making it nearly identical to free space. Both the antenna and the AMCS are affixed to the opposite faces of the foam block using an adhesive material.

### Impedance matching bandwidth measurement for the antenna over AMCS

**Fig. 35:**
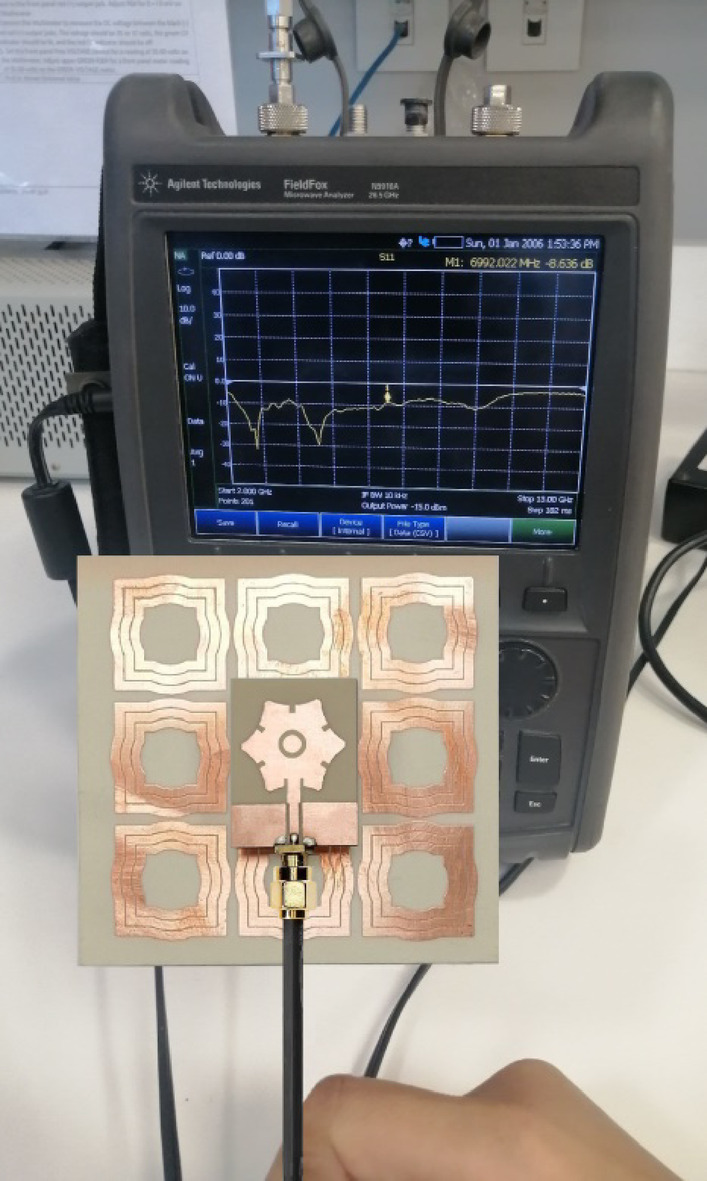
Measurement of $\:{S}_{11}$ using the VNA over the frequency range $2-13\:\text{G}\text{H}\text{z}$. As show, $\:\left|{S}_{11}\right|<-10\:\text{d}\text{B}$ over the frequency range $3.3-7.1\text{G}\text{H}\text{z}$.

**Fig. 36:**
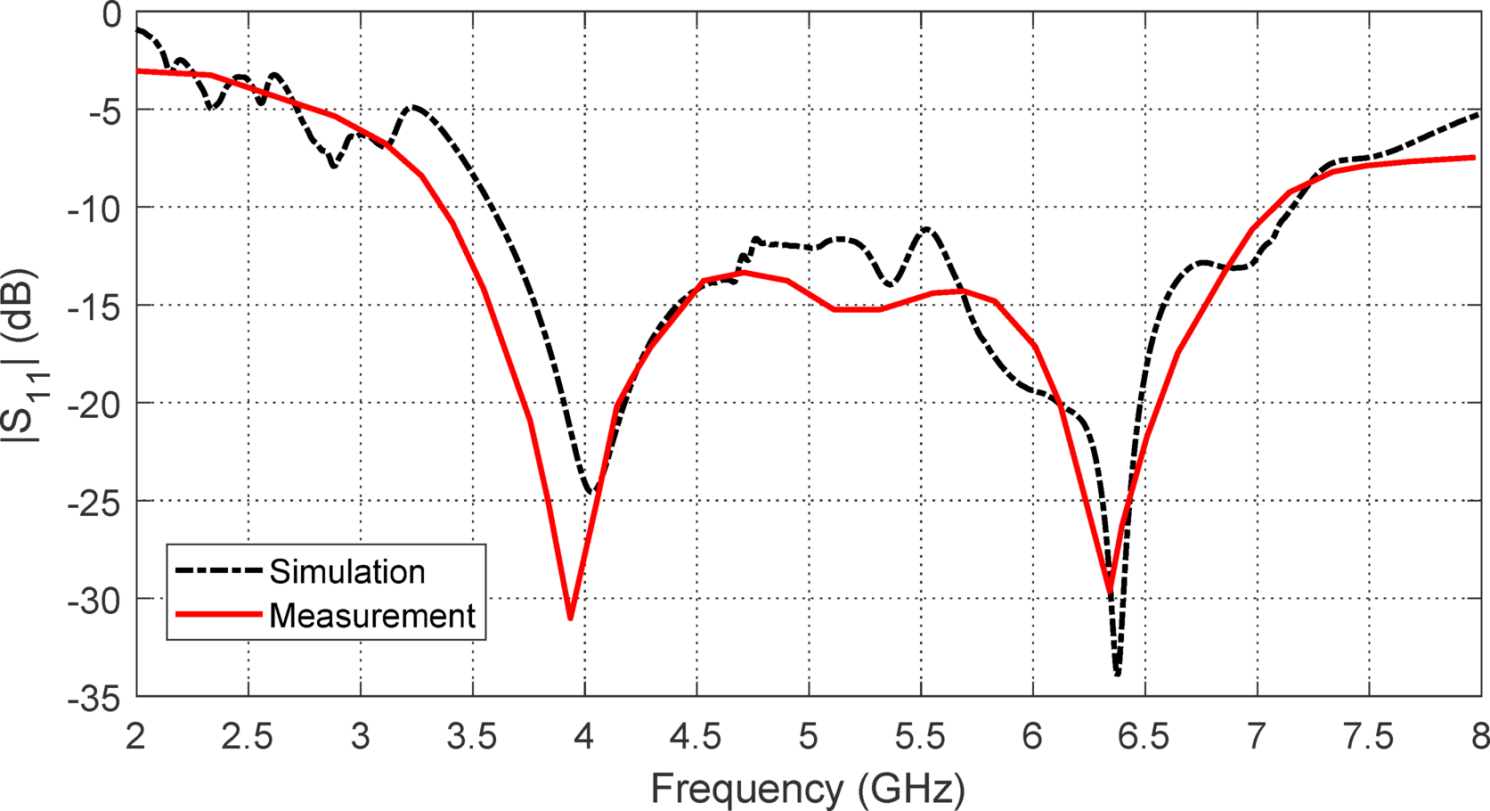
Frequency dependence of $\:\left|{S}_{11}\right|$ over the frequency range $2-8\:\text{G}\text{H}\text{z}$ as obtained by simulation and measurement.

To measure the reflection coefficient at the feeding port of the antenna when backed by the proposed AMC surface, the antenna is connected to port 1 of the VNA (model Agilent N9918 A), as shown in Fig. 35. The measurements are conducted over a frequency range of $2-13$ GHz. The measured magnitude of $\:{S}_{11}$ is plotted and compared with the corresponding simulation results in Fig. 36, showing good agreement. The impedance matching frequency band of the wideband antenna, when backed by the AMC surface at a distance of $\:17.5$ mm, is $3.7-7.2$ GHz based on simulation results, and $3.3-7.1$ GHz based on the measurements. This indicates that positioning the AMC surface at this distance behind the antenna does not negatively affect its impedance matching bandwidth.

### Measurement of the gain of the antenna over the AMC

**Fig. 37:**
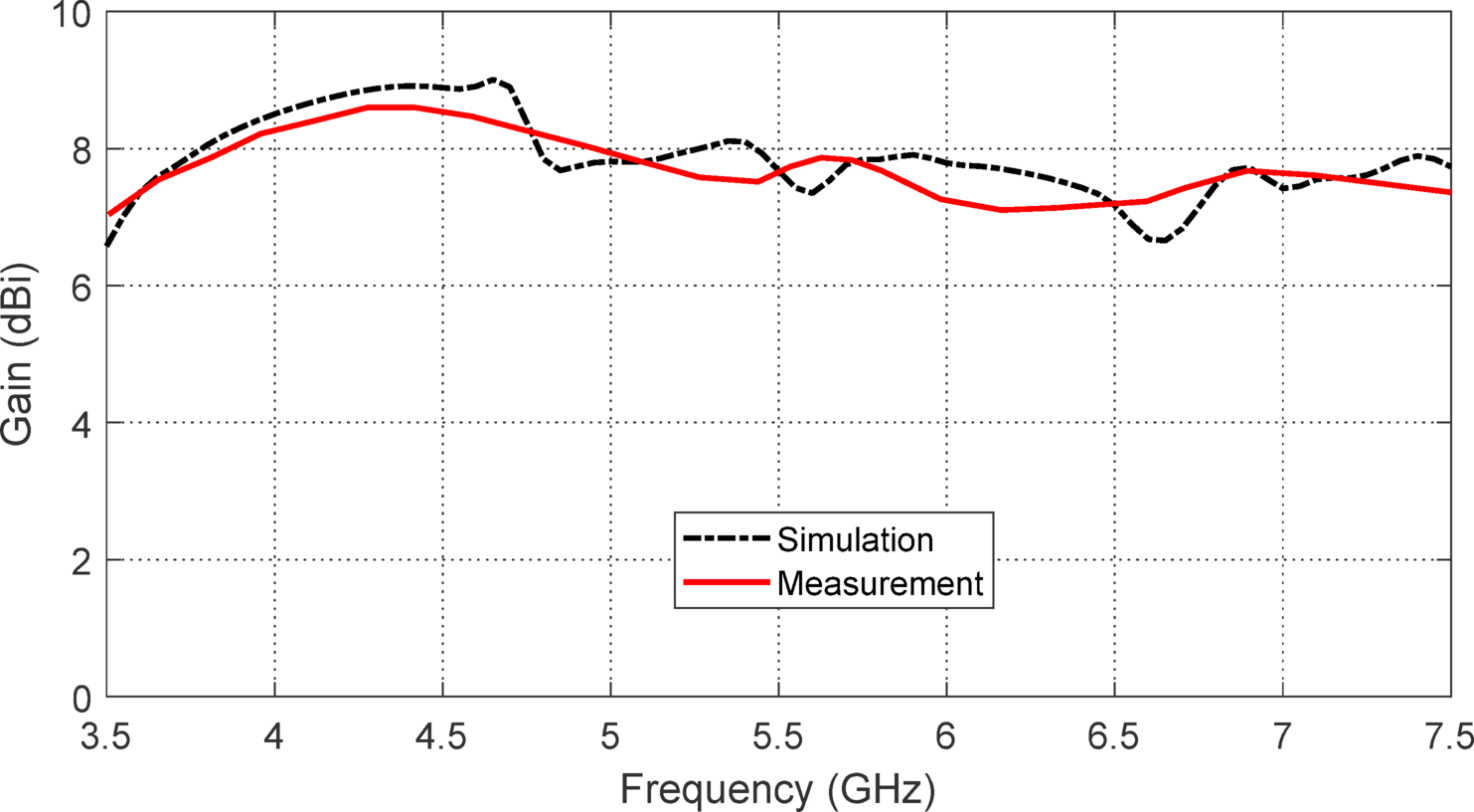
Frequency dependence of the maximum gain of the star-shaped antenna when placed at a height of $\:H=7.5\:\text{m}\text{m}$ above the AMCS.

The gain of the wideband antenna, when backed by the proposed AMC, is measured using the method described in Sect. 4.3. Figure 37 shows the variation of the measured gain with frequency over the impedance matching band, compared to the corresponding simulation results. The measured and simulated gain variations with frequency are found to be in good agreement.

## Comparison with other AMCS-backed antenna designs

To provide a more comprehensive evaluation of the AMCS-backed antenna proposed in this work, its performance in terms of dimensions, gain, and frequency band is compared to the relevant parameters of other antenna designs from recent publications. These comparisons are summarized in Table 3. Among the designs listed, the antenna proposed here offers the widest impedance matching frequency band (70%) and AMCS bandwidth (49%). However, it is relatively larger in size compared to other designs, such as those presented in^8 and 19^.

**Table 3:** Comparison with recently published designs of AMCS-based antennas.

Work	Dimension	Impedance Matching BW (%)	AMC BW (%)	Gain (dBi)
[8]	$\:0.536{\lambda}_{0}\times0.536{\lambda}_{0}$	$2.2-2.65\:\text{G}\text{H}\text{z}\:(18\%)$	$\:5.6\:\%$	$\:4.8$
[19]	$\:0.79{\lambda}_{0}\times0.79{\lambda}_{0}$	$2.37-2.50\:\text{G}\text{H}\text{z}\:(5.34\%)$	$\:15\:\%$	$\:10.3$
[20]	$\:1.2{\lambda}_{0}\times1.2{\lambda}_{0}$	$4-7\:\text{G}\text{H}\text{z}\:(54.54\:\%)$	$\:9\:\%$	$\:10$
[21]	$\:1.23{\lambda}_{0}\times1.13{\lambda}_{0}$	$4.2-8.1\:\text{G}\text{H}\text{z}\:(63\:\%)$	$\:32\:\%$	$\:7.9$
[22]	$\:{\lambda}_{0}\times{\lambda}_{0}$	$1.8-2.7\:\text{G}\text{H}\text{z}$ (36%)	3%	7.2
[23]	$\:{0.56\lambda}_{0}\times{0.56\lambda}_{0}$	$2.25-2.66$ GHz (17%)	18%	8.2
[Present]	$\:1.1{\lambda}_{0}\times1.1{\lambda}_{0}$	$3.6-7.2\:\text{G}\text{H}\text{z}\:(70\%)$)	49%	$\:8.5$

Note: $\:{\lambda\:}_{0}$ corresponds to the lowest operating frequency of the antenna.

## Conclusion

A wideband planar monopole patch antenna has been developed as an omnidirectional antenna, achieving excellent impedance matching and radiation efficiency exceeding $\:98\%$. Its gain ranges from $\:2$ dBi to $\:4.5$ dBi across the frequency range of $3.6-7.2$ GHz when operating in free space. To enhance the antenna’s gain over its operational frequency range, a compact AMCS has been introduced. The AMCS-based antennas are required for many applications including 5G and future wireless networks, fixed satellite services (FSS), mobile satellite communication, Airborne and Ground-based Radar, and automotive radar (for autonomous vehicles). The proposed AMCS consists of only $\:3\times\:3$ unit cells, with overall dimensions of $\:9\times\:9$ cm.

($\:1.1{\lambda\:}_{0}\times\:1.1{\lambda\:}_{0}$). It is designed to be positioned parallel to the planar antenna at a distance of $\:1.75$ cm behind it. The AMCS-backed antenna demonstrates an improved gain of up to 9 dBi while maintaining the impedance matching bandwidth. Additionally, the radiation efficiency remains above $\:98\%$ throughout the operational frequency band of $3.7-7.2$ GHz. For practical evaluation, both the antenna and AMCS were fabricated, and their performance was assessed through measurements of impedance matching bandwidth, gain, and radiation efficiency. The experimental results align closely with the simulation outcomes, validating the proposed design.

## Data Availability

The datasets used and/or analyzed during the current study available from the corresponding author on reasonable request.

## References

[1] Upadhyaya, T. K., Kosta, S. P., Jyoti, R. & Palandöken, M. Novel stacked µ-negative material-loaded antenna for satellite applications. *Int. J. Microw. Wirel. Technol.* **8** (2), 229–235 (2016).

[2] Upadhyaya, T. K., Kosta, S. P., Jyoti, R. & Palandoken, M. Negative refractive index material-inspired 90-deg electrically Tilted ultra wideband resonator. *Opt. Eng.* **53** (10), 107104–107104 (2014).

[3] Jiang, Z., Wang, Z., Nie, L., Zhao, X. & Huang, S. A low-profile ultrawideband slotted dipole antenna based on artificial magnetic conductor. *IEEE Antennas. Wirel. Propag. Lett.* **21**, 4, pp.671–6752022 .

[4] Al-Gburi, A. J. A., Ibrahim, I., Zeain, M. Y. & Zakaria, Z. Compact size and high gain of CPW-fed UWB strawberry artistic shaped printed monopole antennas using FSS single layer reflector. *IEEE Access.* **8**, 2697–2707 (2020).

[5] Pandit, V. K. & Harish, A. R. Compact wide band directional antenna using cross-slot artificial magnetic conductor (CSAMC). *Int. J. RF Microw. Computer‐Aided Eng.*, 4, p.e21577, (2019).

[6] .Wang, W. Y. H., .Che, W. & .Wang, J. A. Wideband and high-gain edge fed patch antenna and array using artificial magnetic conductor structures. *IEEE Antennas Wirel. Propag. Lett.* **12**, pp.769–7722013 .

[7] A.Vallecchi, J. R., .De Luis, F., Capolino, F. D. & .Flaviis Low profile fully planar folded dipole antenna on a high impedance surface. *IEEE Trans. Antennas Propag. Vol*. **60**, 51–62 (2012).

[8] Raad, H. R., Abbosh, A. I., Al-Rizzo, H. M. & Rucker, D. G. Flexible and compact AMC based antenna for telemedicine applications. *IEEE Trans. Antennas Propag.* **61**, 524–531 (2013).

[9] Kumar, R. & Dhubkarya, D. C. UWB compact microstrip patch antenna with high directivity using novel star-shaped frequency selective surface. *Progress Electromagnet. Res. C*. **119**, 255–273 (2022).

[10] Joshi, A. & Singhal, R. Gain enhancement in probe-fed hexagonal ultra-wideband antenna using AMC reflector. *J. Electromagn. Waves Appl.* **33** (9), 1185–1196 (2019).

[11] Mersani, A., Osman, L. & Ribero, J. M. Flexible UWB AMC antenna for early stage skin cancer identification. *Progress Electromagnet. Res. M*. **80**, 71–81 (2019).

[12] Kundu, S., Chatterjee, A., Jana, S. K. & Parui, S. K. A compact umbrella-shaped UWB antenna with gain augmentation using frequency selective surface. *Radio Eng.* **27** (2), 448–454 (2018).

[13] Gao, G. P., Yang, C., Hu, B., Zhang, R. F. & Wang, S. F. A wearable PIFA with an all-textile metasurface for 5 GHz WBAN applications, IEEE Antennas and Wireless Propagation Letters, Vol. 18, No. 2, 288–292, Feb. (2019).

[14] Zhu, J., Li, S., Liao, S. & Xue, Q. Wideband low-profile highly isolated MIMO antenna with artificial magnetic conductor, IEEE Antennas and Wireless Propagation Letters, Vol. 17, No. 3, 458–462, Mar. (2018).

[15] Abdulhasan, R. A., Alias, R., Ramli, K. N., Seman, F. C. & Abd-Alhameed, R. A. High gain CPW-fed UWB planar monopole antenna-based compact uniplanar frequency selective surface for microwave imaging. *Int. J. RF Microw. Comput. -Aided Eng.*, **29**, 8, Art. no. e21757, 2019.

[16] Kumari, P., Gangwar, R. K. & Chaudhary, R. K. An aperture-coupled stepped dielectric resonator UWB MIMO antenna with AMC. *IEEE Antennas. Wirel. Propag. Lett.*, **21**, 10, 2040–2044, Oct. 2022.

[17] Zhang, Y., Li, Y., Zhang, W., Zhang, Z. & Feng, Z. Omnidirectional antenna diversity system for high-speed onboard communications. *Engineering* **11** (4), 72–79 (2022).

[18] Guthi, S. Damera,High gain and broadband circularly polarized antenna using metasurface and CPW fed L-shaped aperture. *AEU-Int J. Electron. Commun. Vol*. **142**, 1–8 (2022).

[19] .Vardaxoglou, J. J. J. C. & Whittow, W. G. Odendaal CPW-fed cavity backed slot radiator loaded with an AMC reflector. *IEEE Trans. Antennas Propag.* **60**, 735–742 (2012).

[20] Prakash, P., Abegaonkar, M. P., Basu, A. & Koul, S. K. Gain enhancement of a CPW-fed monopole antenna using polarization-insensitive AMC structure. *IEEE Antennas Wirel. Propag. Lett. 12*, pp.1315–1318, (2013).

[21] C.Joshi, A. C., Lepage, J., Sarrazin, X. & Begaud Enhanced broadside gain of an ultrawideband diamond dipole antenna using a hybrid reflector. *IEEE Trans. Antennas Propag. 64*, pp.3269–3274,2016.

[22] Ayd, R., Saad, Ayman, W. M., Hassan & Ahmed, A. Ibrahim. A monopole antenna with cotton fabric material for wearable applications. *Sci. Rep.* **13** (1), 7315 (2023). 10.1038/s41598-023-34394-3 PMC10163037 37147522

[23] Rajavel, V. & Ghoshal, D. A compact triband antenna using artificial magnetic conductor for wireless body area network communications front. *Wireless Netw.* **29** (6), 2773–2795 (2023).

